# The Yeast Environmental Stress Response Regulates Mutagenesis Induced by Proteotoxic Stress

**DOI:** 10.1371/journal.pgen.1003680

**Published:** 2013-08-01

**Authors:** Erika Shor, Catherine A. Fox, James R. Broach

**Affiliations:** 1 Department of Molecular Biology, Princeton University, Princeton, New Jersey, United States of America; 2 Department of Biomolecular Chemistry, School of Medicine and Public Health, University of Wisconsin, Madison, Wisconsin, United States of America; 3 Department of Biochemistry and Molecular Biology, Penn State College of Medicine, Hershey, Pennsylvania, United States of America; Duke University, United States of America

## Abstract

Conditions of chronic stress are associated with genetic instability in many organisms, but the roles of stress responses in mutagenesis have so far been elucidated only in bacteria. Here, we present data demonstrating that the environmental stress response (ESR) in yeast functions in mutagenesis induced by proteotoxic stress. We show that the drug canavanine causes proteotoxic stress, activates the ESR, and induces mutagenesis at several loci in an ESR-dependent manner. Canavanine-induced mutagenesis also involves translesion DNA polymerases Rev1 and Polζ and non-homologous end joining factor Ku. Furthermore, under conditions of chronic sub-lethal canavanine stress, deletions of Rev1, Polζ, and Ku-encoding genes exhibit genetic interactions with ESR mutants indicative of ESR regulating these mutagenic DNA repair processes. Analyses of mutagenesis induced by several different stresses showed that the ESR specifically modulates mutagenesis induced by proteotoxic stress. Together, these results document the first known example of an involvement of a eukaryotic stress response pathway in mutagenesis and have important implications for mechanisms of evolution, carcinogenesis, and emergence of drug-resistant pathogens and chemotherapy-resistant tumors.

## Author Summary

Cellular capability to mutate its DNA plays an important role in evolution and impinges on medical issues, including acquisition of mutator phenotypes by cancer cells and emergence of drug-resistant pathogens. Whether and how the environment affects rates of mutation has been studied predominantly in the context of environmental agents that damage DNA (e.g. UV and γ-rays). However, it has been observed that conditions of chronic non-DNA-damaging stress (e.g. starvation or heat shock) also increase mutagenesis. It has been shown that in bacteria, activation of the general stress response activates a pro-mutagenic pathway and thus promotes mutagenesis during periods of stress. However, in eukaryotes, so far there has been no evidence of a stress response regulating mutagenesis. In this manuscript we demonstrate that in budding yeast, a model eukaryote, the general environmental stress response (ESR) regulates mutagenesis induced by proteotoxic stress (accumulation of unfolded proteins) at several loci. We also identify two pro-mutagenic DNA metabolic pathways that contribute to this mutagenesis and present genetic data showing that the ESR regulates these pathways. Together, these data advance our understanding of how cellular sensing and responding to environmental cues affect cellular capability for mutagenesis.

## Introduction

Sensing and responding to environmental cues are ubiquitous cellular functions essential for survival. Budding yeast cells respond to a variety of stresses by inducing or repressing specific sets of genes in a stereotypical fashion that, to a certain degree, does not depend on the identity of the stress. This process is termed the environmental stress response (ESR) [1], [2]. Paradoxically, the ESR provides little protection from the initiating stress – genes required to survive the stress do not significantly overlap those that change expression in response to the stress and mutations in ESR regulators do not significantly sensitize cells to stress [3], [4]. This observation raises the possibility that ESR activation may have other cellular roles. One potential role of the ESR is suggested by observations that chronic stress can induce genetic instability in different organisms [5]–[10]. The phenomenon of stress-associated genetic instability impinges on medical issues, such as the role of tumor microenvironment in genetic instability of cancer cells and emergence of drug-resistant pathogens and chemotherapy-resistant tumors. While in *Escherichia coli* stress response can activate mutagenic DNA repair [11], [12], no evidence exists as yet for the involvement of the ESR in mutagenesis in a eukaryote. In this manuscript we investigate the effects of stress on mutagenesis in yeast and the role of the ESR in this process.

Numerous studies have indicated that environmental stress can affect genome stability. For instance, in mammalian cells in tissue culture hypoxia and starvation can suppress error-free DNA repair pathways (e.g. mismatch repair and homologous recombination) and cause an increase in mutagenesis [13]–[19]. In yeast, various types of stress can affect chromosome segregation and promote aneuploidy [6]. Interestingly the most potent inducer of aneuploidy is proteotoxic stress, e.g. inhibition of HSP90 protein chaperone by radicicol [6]. One explanation of this phenomenon is that HSP90 can become “overtaxed”, such that its client proteins that function in chromosome segregation would interact with their targets in a misfolded, disfunctional state, with aberrant consequences for ploidy maintenance [6]. Other instances of genetic instability, in particular mutagenesis, were reported in response to chronic osmotic and DNA replication stresses [20], [21]. These types of stress are thought to be mutagenic at least in part because they can directly cause DNA damage: osmotic stress induces DNA breaks [22] and replication stress stalls DNA replication forks and creates regions of ssDNA [23]. Finally, several groups reported the phenomenon of “adaptive mutation” (alternately termed “stationary phase” or “selection-induced” mutation) in budding yeast *Saccharomyces cerevisiae* ([9], [10]; for a comprehensive review, see [24]). In these experiments, starvation for an amino acid induced reversions of mutations in amino acid biosynthesis genes, enabling cells to grow on the starvation medium. Besides amino acid starvation, “adaptive” mutants were also observed after exposure of yeast cells to the drug canavanine [25]. Together these studies suggest that sensing and responding to environmental stress may have important consequences for genome stability, but mechanisms underlying this assertion and the involvement of stress responses in these phenomena remain underexplored.

Cellular pathways that function in mutagenesis in eukaryotes have been extensively studied, predominantly by identifying mutants defective in spontaneous and/or DNA damage-induced mutagenesis. These analyses have identified DNA translesion synthesis (TLS) as a key mutagenic pathway in both yeast and higher eukaryotes [26]. In yeast, TLS is largely carried out by two specialized DNA polymerases Rev1 and polymerase ζ (Polζ) that, unlike replicative polymerases δ and ε, can polymerize DNA using damaged or distorted DNA templates and thus function in DNA damage bypass pathways [27]. While Rev1 and Polζ can interact and function together in vivo, they do not have identical phenotypes in all mutation assays, suggesting that they have some independent roles [26]. In contrast to spontaneous and DNA damage-induced mutagenesis, genetic requirements for stress-associated mutagenesis are less well characterized. However, Heidenreich *et al.* reported that starvation-associated frameshift reversion was independent of both Rev1 and Polζ [28], instead requiring proteins that function in non-homologous end joining (NHEJ), such as Ku [29]. NHEJ is a DNA double-strand break (DSB) repair pathway that directly ligates broken ends together without relying on a homologous template [30]. Whether these mutagenic repair pathways are important for other types of stress-associated mutagenesis and are influenced by cellular stress responses has not yet been examined.

The ESR in *S. cerevisiae* is activated in response to any one of a large number of environmental stresses [1], [2], including DNA damaging agents, such as the DNA alkylating drug methylmethane sulfonate and inhibitor of ribonucleotide reductase hydroxyurea [31]. The genes that are repressed by the ESR largely function in translation and other growth-promoting pathways. The genes that are induced by the ESR function in several molecular processes, such as protein folding and repair of oxidative damage, but the functions of many of them are not known. Stress-driven induction of most ESR-activated genes is largely regulated by partially redundant transcription factors, Msn2 and Msn4 [1], [2], [32], [33]. Of the two proteins, Msn2 plays a greater role in transcriptional activation and its behavior has been relatively well examined. In unstressed cells, Msn2 is localized almost exclusively to the cytoplasm. Upon a sudden stress, such as a drop in glucose concentration or osmotic shock, Msn2 moves into the nucleus in the majority of cells where it binds to stress response elements in its targets' promoters to activate transcription [34]–[36].

In this manuscript, we examine the effect of the ESR on mutagenesis in *S. cerevisiae* by analyzing spontaneous and stress-associated mutagenesis in mutants lacking *MSN2* and *MSN4*. We report that the drug canavanine causes proteotoxic stress and activates the ESR, and that under conditions of severe canavanine stress *MSN2* and *MSN4* promote certain types of mutation events, most notably single nucleotide deletions in simple repeats. Furthermore, while *MSN2* and *MSN4* are dispensable for mutagenesis induced by osmotic and DNA replication stresses, they can promote or suppress mutagenesis induced by different types of proteotoxic stress. Furthermore, TLS polymerases and Ku also function in proteotoxic stress-induced mutagenesis and exhibit unanticipated genetic interactions with *MSN2* and *MSN4*. Together, these results implicate the yeast ESR in regulation of mutagenic DNA repair pathways activated by proteotoxic stress.

## Results

### Canavanine promotes mutagenesis of *CAN1*

Canavanine is a toxic analog of arginine and can be imported into yeast cells via an arginine transporter, Can1. The *CAN1* gene is a commonly used mutation reporter in yeast as *can1* mutations can be selected on plates containing canavanine. In a typical experiment, *CAN1* cultures grown in the absence of canavanine are plated on canavanine-containing plates, so that only *can1* mutants can form colonies. The mutation rates are then calculated from the *can1* colony distribution data [25], [37]. If *can1* mutants form spontaneously during cell division in culture, the frequency of mutants should follow a Luria-Delbrück distribution [38]. An earlier study by Lang and Murray, designed to accurately calculate *CAN1* mutation rate in culture using a large-scale fluctuation assay, detected a significant deviation of the data from a Luria-Delbrück distribution, suggesting that some *can1* mutants were forming *after* plating the cultures on canavanine plates [25]. Raising the concentration of canavanine ten-fold (to 600 µg/ml) decreased, but did not eliminate, post-plating mutation. Using this high canavanine concentration and excluding small colonies from the calculation led to better fit of the data to a Luria-Delbrück distribution, suggesting that small colonies were largely *can1* mutations that occurred after plating.

**Figure 1:**
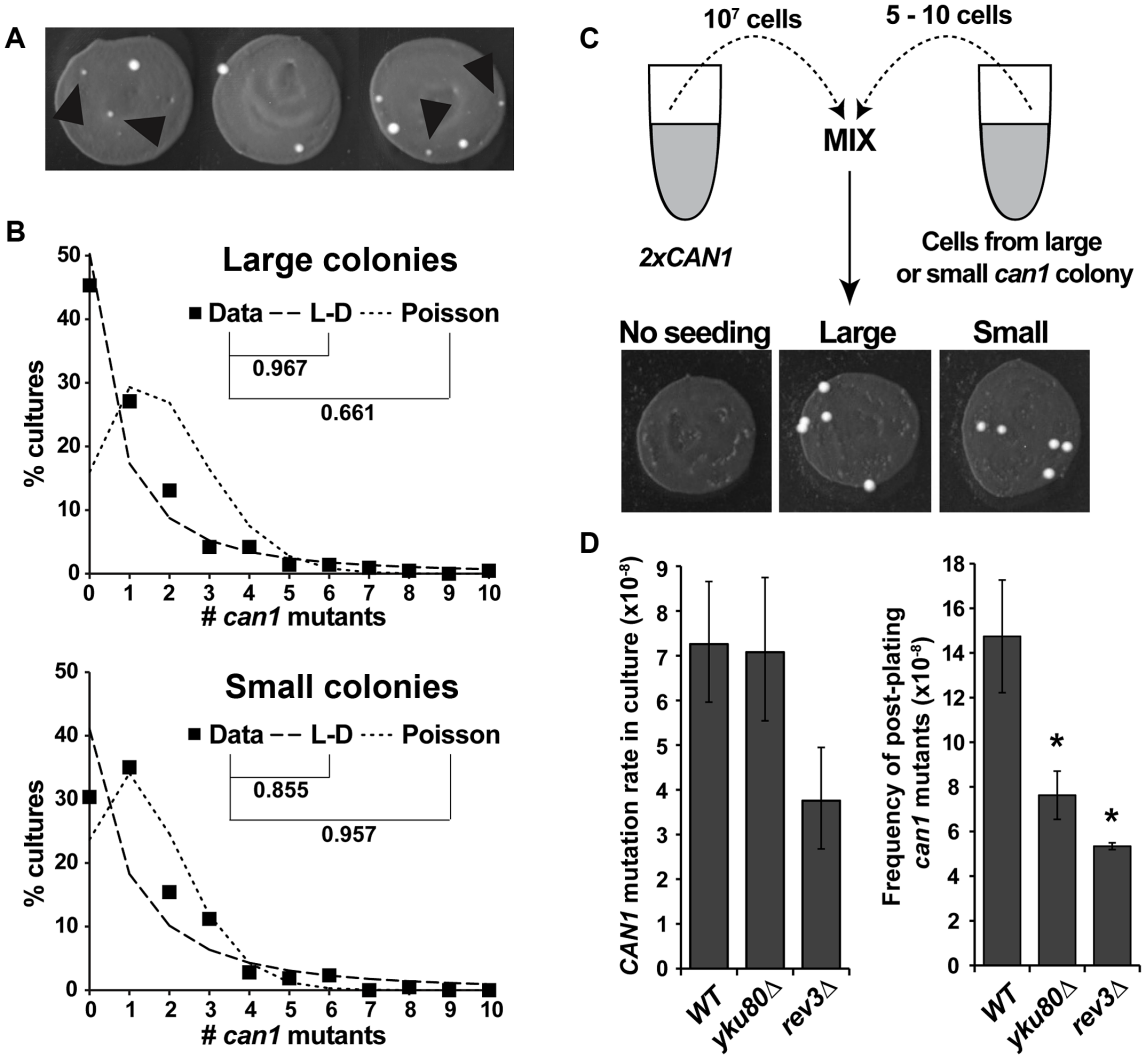
*CAN1* mutations can occur after cells are plated on canavanine medium. (A) Cultures incubated on canavanine plates produced *can1* mutant colonies, some of which were very small (black arrowheads). (B) “Large” colony data from >200 independent cultures fit Luria-Delbrück distribution (L-D) better than Poisson distribution, while “small” colony data fit Poisson better than L-D. Correlation coefficients between data and theoretical distributions that best fit the data are shown. (C) Reconstruction experiment demonstrating that small canavanine-resistant colonies are not inherently slow growing. All of the *can1* colonies originated from seeded *can1* cells and all gave rise to large colonies, irrespective of whether they originally came from large or small *can1* colonies. (D) NHEJ and DNA polymerase ζ affect canavanine-induced mutagenesis. *yku80Δ* reduced post-plating mutation (right panel) while leaving mutation in culture unaffected (left panel), while *rev3Δ* reduced both *CAN1* mutation in culture and post-plating. Error bars represent 95% confidence intervals for mutation rates in culture and standard deviations for post-plating mutation frequencies (*, P<0.05, Student's t-test).

We used the same large-scale fluctuation assay and also obtained evidence for post-plating *can1* mutation. Similar to Lang and Murray, we found that eliminating small colonies (Figure 1A) from the dataset improved the fit of the data to a Luria-Delbrück distribution. The frequency of large colonies exhibited a better fit to a Luria-Delbrück distribution than to a Poisson distribution, while small colonies fit a Poisson distribution better than a Luria-Delbrück distribution (Figure 1B), consistent with the conclusion that small colonies were more likely to have arisen after selection had been imposed. We performed a reconstruction experiment to investigate the possibility that small colonies were simply inherently slower growing than the large colonies (Figure 1C). Six large and six small independent *can1* mutants were picked, purified and seeded into a culture of a carrier strain that harbored two copies of the *CAN1* gene, and whose rate of canavanine resistance was accordingly negligible relative to the experimental strains. The carrier cultures were then plated on canavanine-containing medium. We observed that all seeded cells, whether they came from original large or small *can1* colonies, formed large colonies in the reconstruction experiment (Figure 1C), ruling out any general or context-specific growth defects for cells in small colonies. In sum, the statistical modeling and the reconstruction experiment strongly supported the conclusion that the majority of small colonies were due to post-plating *can1* mutations.

The only pathway known to promote “adaptive” mutagenesis in previously reported reversion assays is non-homologous end joining (NHEJ) [29]. To investigate the role of NHEJ in post-plating *can1* mutation, we deleted *YKU80* and observed that our *yku80Δ* strain exhibited an approximately two-fold reduction in the frequency of post-plating *can1* mutation without any change in *CAN1* mutation rate during mitotic growth (Figure 1D). We also tested the involvement of a TLS DNA polymerase, pol ζ, by deleting its subunit *REV3*. The *rev3Δ* mutant exhibited reduced *CAN1* mutation frequency both in culture (two-fold) and after plating (2.75-fold). Overall, these results demonstrated that NHEJ and TLS help promote *CAN1* mutagenesis during acute canavanine exposure.

### Post-plating *can1* mutations exhibit a distinct mutation spectrum

**Figure 2:**
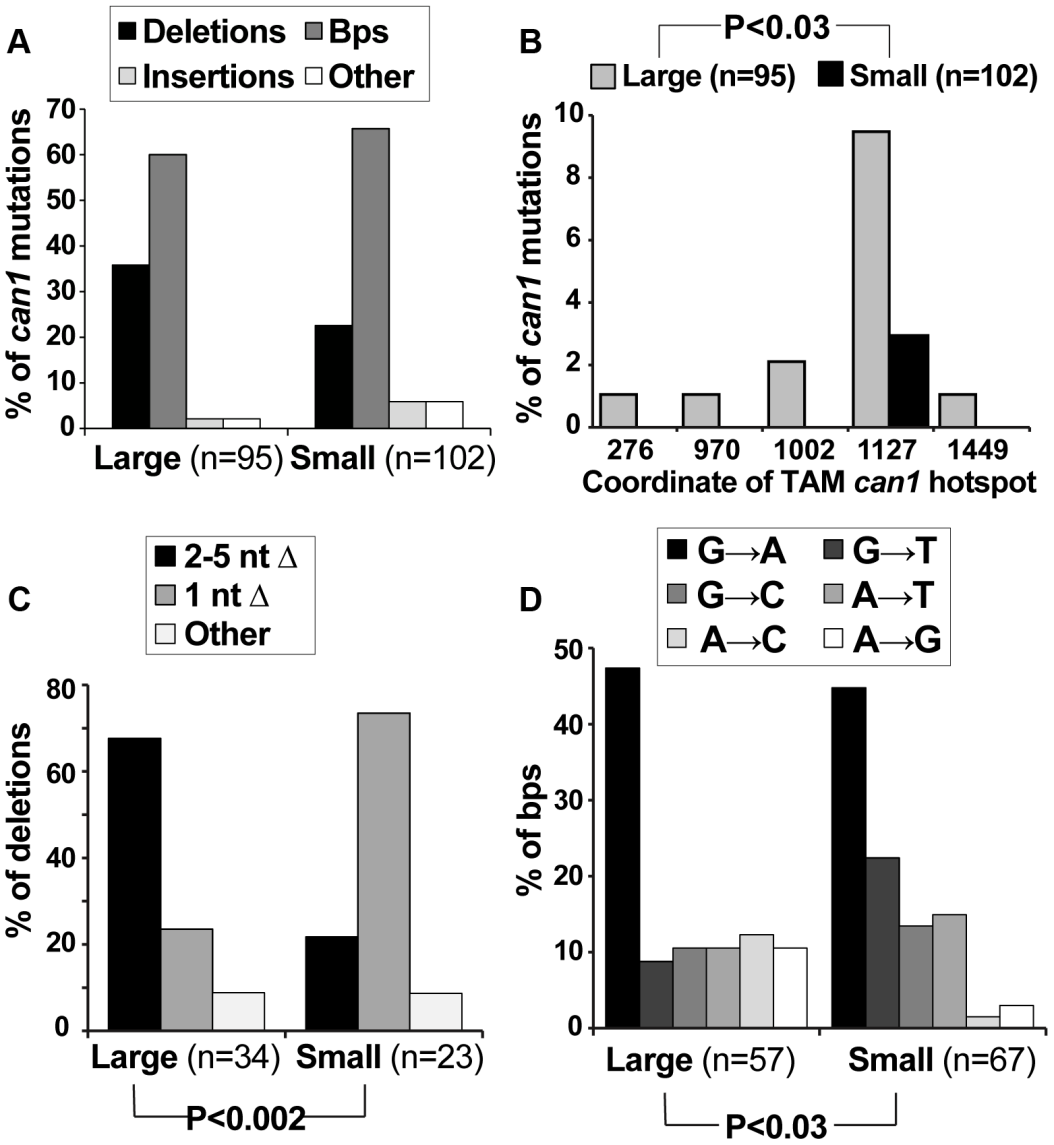
Mutation spectra of *can1* mutants generated on canavanine plates exhibit several significant differences with those generated in culture. (A) The overall distribution of different *can1* mutation categories is not altered between pre-plating(large) and post-plating (small) *can1* mutants. The “other” category contains multiple mutations per *can1* ORF, usually clustered together. (B) Pre-plating (large) but not post-plating (small) *can1* mutants contained transcription-associated mutation (TAM) hotspots. Lippert et al identified the five main TAM hotspots in *CAN1*, with ATΔ at CATAT at position 1127 being the most frequent in both their study and ours [39]. (C) Post-plating mutants were characterized by a significant increase in single nucleotide deletions (predominantly occurring in mononucleotide runs; Table S1). (D) Base pair substitution spectra differed significantly between pre- and post-plating mutants.

We speculated that if small *can1* colonies arose under different conditions than large colonies (i.e. during acute exposure to canavanine on plates), they might have been generated by different mutagenesis mechanisms and thus might have different mutation spectra. Therefore, we sequenced the *can1* ORF from a large number of pre-plating (large) and post-plating (small) *can1* colonies. All of the sequenced alleles contained at least one mutation in the *CAN1* ORF (Table S1). The predominant classes of mutations were deletions and base pair substitutions, and the overall distribution of these broadly defined mutation classes was not significantly different between large and small colonies (Figure 2A). However, finer grained analysis of the sequence data yielded several significant results. First, a significant proportion of mutations in large *can1* colonies occurred at sites previously identified as hot-spots of transcription-associated mutation (TAM) in *CAN1* [39]. For example, ATΔ at position 1127 was previously identified as the most frequent mutation in actively transcribed *CAN1* [39] and was, in this study, the single most frequent mutation in the large *can1* colonies (Figure 2B; Table S1). This result showed that *can1* mutations in large colonies were generated in cells actively transcribing *CAN1*, consistent with our earlier conclusion that these mutations occurred in actively growing cells in culture. In contrast, *can1* mutations in small colonies did not exhibit a TAM signature, indicating that these mutations occurred under conditions of low transcriptional activity and consistent with the hypothesis that they arose after plating. Second, small colonies differed significantly from large ones (Fisher exact test P<0.002) with respect to the types of deletions in *CAN1*. In particular, the majority of deletions in large colonies were those of 2–5 nucleotides, while over 70% of deletions in small WT colonies were those of a single nucleotide (Figure 2C; Table S1), with 13 out of 17 such −1 deletions occurring in simple repeats (i.e. mononucleotide runs). Finally, we observed a statistically significant difference in the types of base pair substitutions in large versus small colonies (P = 0.028; Figure 2D). Together, these data confirmed our earlier conclusions that *can1* mutations in small colonies arose under different conditions than those in large colonies and were generated by distinct mutagenesis mechanisms.

### The role of the ESR in canavanine-induced mutagenesis

**Figure 3:**
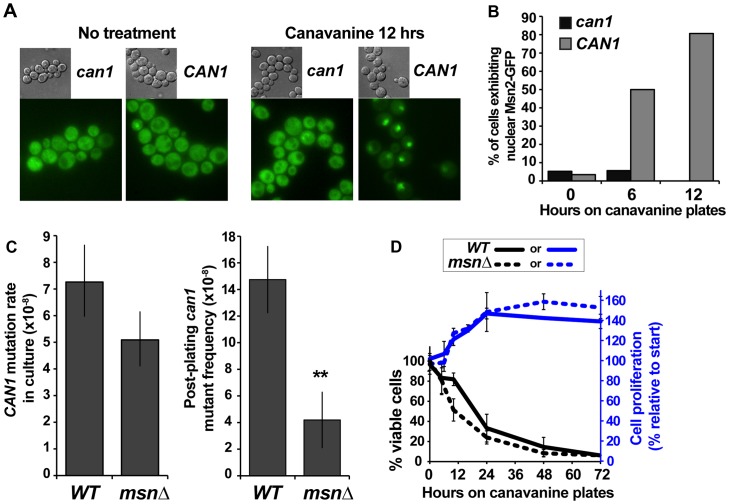
The ESR plays a role in canavanine-induced *can1* mutation. (A) and (B) Nuclear Msn2-GFP localization increased in canavanine-sensitive cells after plating them on canavanine, reflecting ESR activation. For the graph in (B) 20 to 55 cells were analyzed for each condition and the differences between *CAN1* and *can1-100* strains at 6 and 12 hours were highly statistically significant (Fisher exact test P<10^−5^). (C) *msnΔ* greatly reduced post-plating *CAN1* mutation (**, P = 0.005, Student's t-test; averages and standard deviations of three experiments are shown) but not mutation in culture (calculated from L-D distributions of large *can1* mutants; error bars show 95% confidence intervals). (D) Cell survival and proliferation on canavanine medium were not significantly affected by *msnΔ*. Averages and standard deviations of three to nine experiments are shown.

Our results revealed that we had recovered two classes of *can1* mutants – those that arose in culture in absence of exogenous stress and those that arose in cells experiencing acute canavanine toxicity on plates. While other examples of mutagenesis in yeast during stressful conditions (including post-plating mutagenesis on canavanine [25]) have been reported, the roles of stress responses in this mutagenesis have not been examined. We addressed the role of the ESR in mutagenesis on canavanine plates by, first, asking whether canavanine activated the ESR and, second, whether attenuation of the ESR affected post-plating mutagenesis. Re-localization of Msn2 from the cytoplasm to the nucleus is a key marker of ESR activation [35]. Thus, to examine the effect of canavanine on the ESR, we examined the subcellular localization of Msn2-GFP in both *CAN1* and *can1* cells after plating them on canavanine medium. While Msn2-GFP was cytoplasmic in the majority of unstressed cells, it relocated to the nucleus in *CAN1* cells after plating on canavanine, with about 80% of the cells showing nuclear Msn2-GFP by 12 hours after plating (Figure 3A and 3B). In contrast, Msn2-GFP in *can1* cells remained cytoplasmic. This difference was not due to a general defect of *CAN1* cells in responding to stress, as *CAN1* and *can1* cells mount a similar response to glucose starvation (Figure S1). Thus, canavanine treatment activated the ESR in yeast cells.

We next asked whether a functional ESR was required for post-plating mutation on canavanine. *MSN2* and *MSN4* function in a partially redundant manner, so we deleted both genes and measured the effect of the *msn2Δ msn4Δ* mutant (hereafter referred to as *msnΔ*) on *CAN1* mutation in culture and on canavanine plates. Strikingly, and as predicted if ESR is necessary for post-plating mutations, the frequency of post-plating *can1* mutants was reduced over 3-fold in the *msnΔ* strain relative to the *MSN* strain (Figure 3C). In contrast, *CAN1* mutation rate of *msn*Δ cells in culture was only slightly decreased relative to that of *MSN* cells. To test whether reduced frequency of post-plating mutants in the *msnΔ* strain might be simply explained by its reduced proliferation or viability on canavanine, we measured proliferation and viability of both WT and *msnΔ* cells after plating them on canavanine. As expected, acute exposure to the high concentration of canavanine in the plates (600 µg/ml) was lethal to cells, but the onset of lethality was gradual, with about a third of the cells still viable after 24 hours. The *msnΔ* mutants were only slightly more sensitive to canavanine than WT cells (Figure 3D), consistent with previous reports that the *MSN* genes support acquired, rather than primary, stress resistance [3]. Also, for both WT and *msnΔ* strains, cell number increased by approximately 50% between 10 and 24 hrs after plating on canavanine, indicating that at least half of the cells divided during that time (Figure 3D). We also ruled out the possibility that post-plating mutant formation was simply delayed in the *msnΔ* strain by monitoring emergence of new *can1* colonies in 72 WT and 72 *msnΔ* cultures for 15 days after plating. We detected no evidence that post-plating mutations simply arise later in the *msnΔ* strain (Figure S2). Together, these results demonstrated that the difference in post-plating *can1* mutation between WT and *msnΔ* strains could not be attributed to differences in survival, proliferation, or a delay in mutant emergence. We conclude therefore that in the *msnΔ* strain the reduction in post-plating *can1* mutants is due to a defect in mutagenesis.

**Figure 4:**
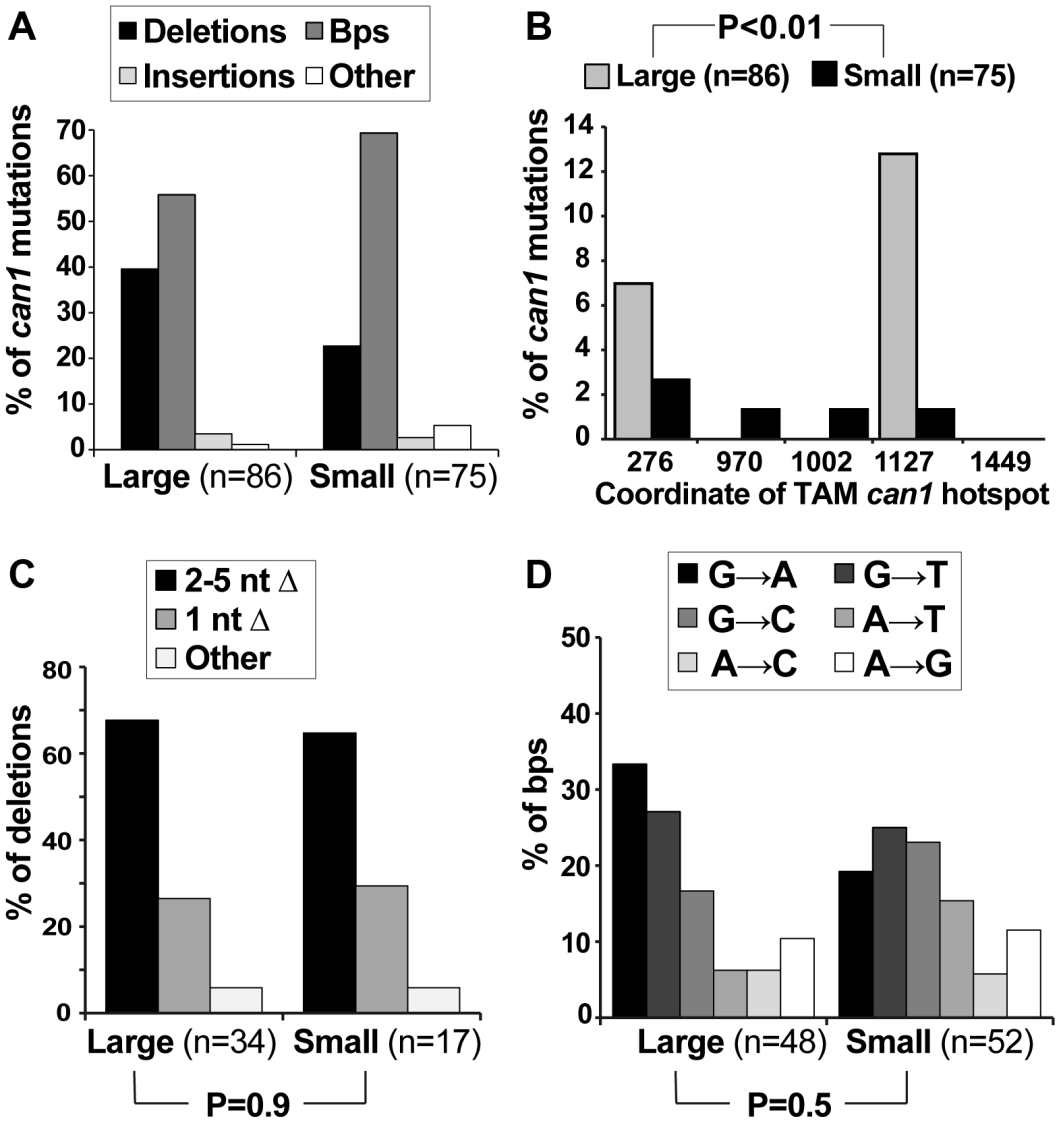
Mutation spectra of pre- and post-plating *can1* mutants generated in the *msnΔ* strain. (A) The overall distribution of different *can1* mutation categories is not altered between pre-plating (large) and post-plating (small) *can1* mutants derived from the *msnΔ* strain. (B) As in the WT strain, pre-plating (large) but not post-plating (small) *can1* mutants derived from the *msnΔ* strain contained transcription-associated mutation (TAM) hotspots [39]. (C) In contrast to the WT strain where there was a significant shift in the types of deletions observed in the large vs small *can1* colonies (Figure 2C), there was no such shift in the *msnΔ* mutant. (D) Also unlike the WT strain, in the *msnΔ* mutant there was no significant change in the types of base pair substitutions recovered from pre- and post-plating *can1* mutants.

If Msn2-Msn4 were important for generating *can1* mutations on canavanine plates but not in culture, we might expect deletion of *MNS2* and *MSN4* to affect specifically the post-plating *can1* mutation spectrum. To address this possibility, we sequenced the *CAN1* ORF in pre-plating (large) and post-plating (small) *can1* mutants generated in the *msnΔ* strain (Figure 4). The resulting mutation spectra had two similarities and two important differences compared to the WT *can1* spectra. First, as in the WT strain, the overall distribution of broadly defined mutation types was not significantly different between large and small *msnΔ* colonies (Figure 4A). Second, as in the WT strain, only large *msnΔ* colonies were significantly enriched for mutations at *CAN1* TAM hot-spots, such as ATΔ at position 1127 (Figure 4B). The observation of TAM hot-spots in large but not small *msnΔ* colonies indicated that, as in the WT strain, the majority of large *msnΔ* colonies were due to mutations that arose in culture, while the majority of small *msnΔ* colonies were due to mutations that arose after plating on canavanine. This conclusion was especially significant given that with regard to deletion and base pair substitution spectra *msnΔ* post-plating mutants were strikingly different from WT post-plating mutants. With respect to deletion types, in contrast to the WT strain, *msnΔ* small colonies showed no shift toward −1 deletions relative to large colonies (Figure 4C). Also unlike the WT strain, where small colonies showed a statistically significantly different base pair substitution spectrum from large colonies, large and small *msnΔ* colonies showed no difference in base pair substitution spectra (Figure 4D). Intriguingly, the unstressed base substitution spectra looked somewhat different for WT and *msnΔ* strains (compare large WT spectra in 2D to *msnΔ* spectra in Figure 4D). This difference was not statistically significant (P = 0.12) but nevertheless suggested that even in the absence of exogenous stress ESR activity may also exert subtle effects on mutagenesis. In sum, we observed that *msnΔ* affected specifically post-plating *can1* deletion and base pair substitution spectra, indicating that the ESR controls specific mutagenesis mechanisms operating in cells exposed to canavanine.

### Canavanine-induced mutagenesis in culture: involvement of the ESR, translesion polymerases, and Ku

**Figure 5:**
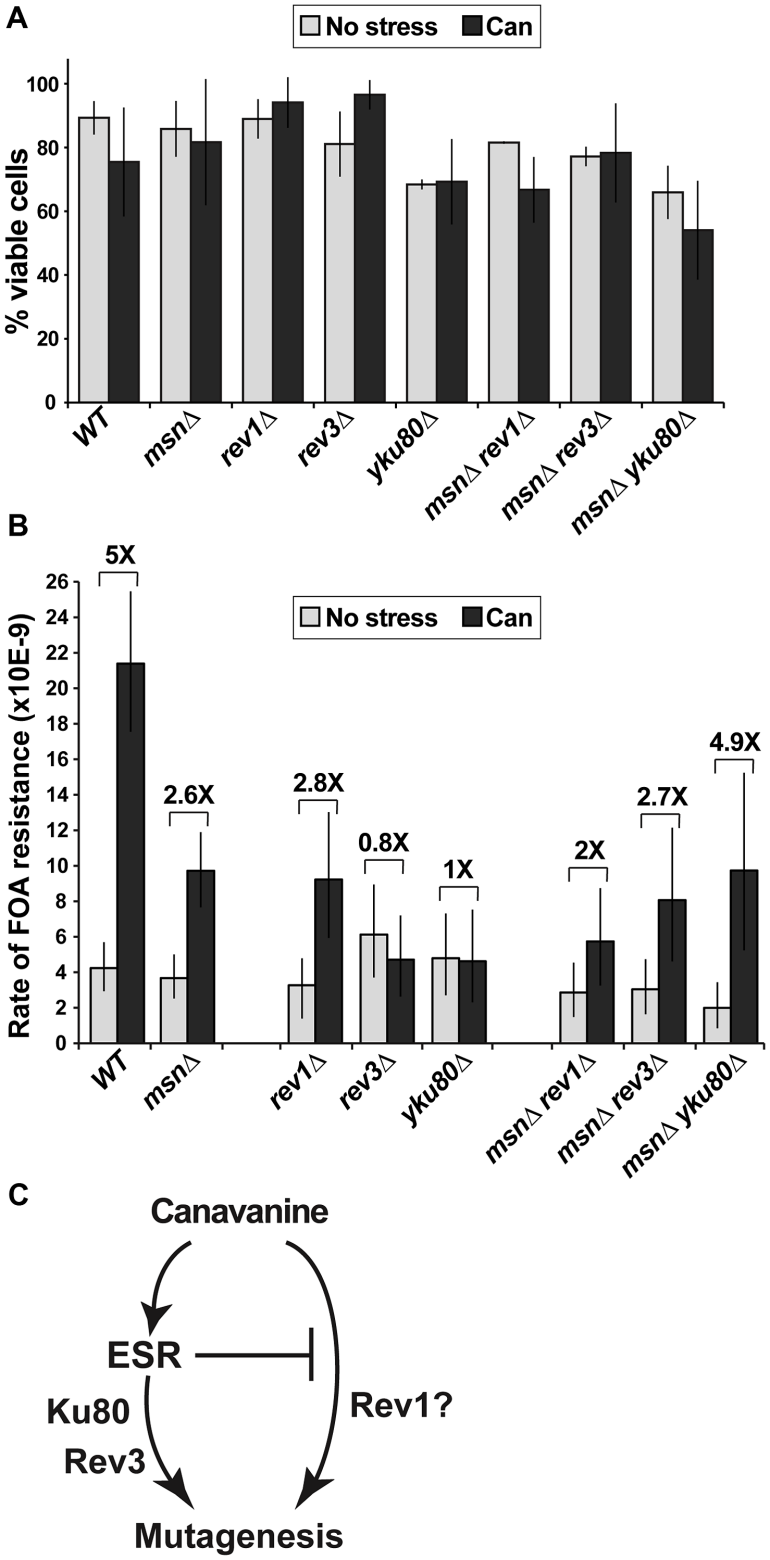
A sub-lethal canavanine concentration induced mutagenesis in culture that involves Msn2-Msn4, Rev1, Rev3 and Ku. (A) 2.5 µg/ml canavanine is not lethal to cells of the various mutants examined in these experiments. Average plating efficiencies and standard deviations of at least three cultures for each strain are shown. (B) Canavanine induces mutagenesis in culture, generating FOA^R^ mutants. Canavanine-induced mutagenesis is partially reduced in strains deleted for *MSN2* and *MSN4* (*msnΔ*) or *REV1* and fully abolished in strains deleted for *REV3* or *YKU80*. Also, *MSN2* and *MSN4* have different genetic interactions with *REV1* and *REV3*/*YKU80* in this process: *rev1Δ* is further compromised for canavanine-induced mutagenesis by *msnΔ*, while *rev3Δ* and *yku80Δ* are rescued by the *msnΔ* mutant. Mutation rates were calculated from 80 to 300 cultures for each genotype; error bars show 95% confidence intervals. (C) A simple model, consistent with data in Figure 5B, proposes that the ESR functions upstream of Rev3 and Yku80 in canavanine-induced mutagenesis.

If canavanine exerted general mutagenic effects, we should be able to detect these effects at other reporter loci, as well as test whether mutagenesis at these loci is affected by the ESR. However, the lethality of *CAN1* cells in the high concentration of canavanine in plates makes it difficult to ask whether mutation at loci other than *CAN1* is also induced after plating. Accordingly, we identified a low concentration of canavanine (2.5 µg/ml) that elicited a stress response, as judged by increased Msn2 nuclear localization (Figure S3A) and slower growth (Figure S3B) but did not affect cell viability (Figure 5A). Then, under conditions of chronic sub-lethal canavanine stress in culture, we measured the rate of forward mutation resulting in resistance to 5-fluoroorotic acid (FOA). In yeast, FOA resistance (FOA^R^) is associated with mutations in the *URA3* gene; however, mutations at other loci can also cause FOA resistance in *URA3* cells [25]. Indeed, we observed that mutations in *URA3* accounted for only a fraction of spontaneous or canavanine-induced FOA^R^ mutants, the rest being comprised of mutations in other, as yet unidentified loci (Figure S4). As with *CAN1*, rates of generation of FOA^R^ mutations were similar in WT and *msnΔ* cells growing in culture in the absence of stress (Figure 5B). The low concentration of canavanine was mutagenic in culture, inducing formation of FOA^R^ mutations by five-fold in WT cells (Figure 5B). Importantly, and as predicted by results obtained with *can1*, this mutagenesis depended in part on Msn2-Msn4, as canavanine induced FOA^R^ in *msnΔ* by only 2.6-fold (Figure 5B). These results showed that the mutagenic effect of canavanine was not limited to *can1* and could be detected at other loci, where it also depended on the function of Msn2-Msn4.

To ask whether mutagenesis by chronic, low-level canavanine exposure also depended on NHEJ and TLS, we analyzed the involvement of Ku, Rev1, and Polζ in this process by deleting *YKU80*, *REV1*, and *REV3* and measuring spontaneous and canavanine-induced rates of FOA^R^ in the mutant strains. We observed that deletion of *REV1* partially reduced canavanine-induced FOA^R^, while deletion of *REV3* or *YKU80* completely abolished it (Figure 5B). Thus, in an otherwise WT background, the functions of Polζ and Ku were required for canavanine-induced mutagenesis. To begin to understand the relationships between the ESR and the DNA repair and DNA damage bypass pathways implicated in canavanine-induced mutagenesis (i.e. TLS and NHEJ), we combined deletions of *MSN2* and *MSN4* with *rev1Δ* or *rev3Δ* or *yku80Δ* and measured the rates of emergence of FOA^R^ in the triple mutants in culture in the absence and presence of canavanine (Figure 5B). Canavanine induced FOA^R^ mutations in the *msnΔ rev1Δ* mutant by about two-fold, showing an attenuation of mutagenesis relative to the *msnΔ* or the *rev1Δ* mutants (Figure 5B). This result indicated that the *msnΔ* and *rev1Δ* mutations had at least partially independent effects on canavanine-induced mutagenesis (Figure 5C). When we measured canavanine-induced mutagenesis in *msnΔ rev3Δ* and *msnΔ yku80Δ* strains, we were surprised to find that, unlike the *rev3Δ* or *yku80Δ* single mutants, the triple mutants were not defective for canavanine-induced mutagenesis, but instead were able to induce mutagenesis in canavanine at least as well as the *msnΔ* mutants did (Figure 5B). Thus, *msnΔ* was epistatic to *rev3Δ* and *yku80Δ* for canavanine-induced mutagenesis. This result suggested that in the presence of low level, chronic canavanine stress the ESR functioned upstream of Rev3 and Yku80 and also suppressed another mutagenic pathway, potentially one involving Rev1 (Figure 5C). Together, these results showed that the ESR could either promote or suppress pro-mutagenic pathways and linked the ESR and error-prone DNA repair and DNA damage bypass pathways.

### Mutagenesis induced by osmotic or DNA replication stress does not depend on *MSN2* and *MSN4*

**Figure 6:**
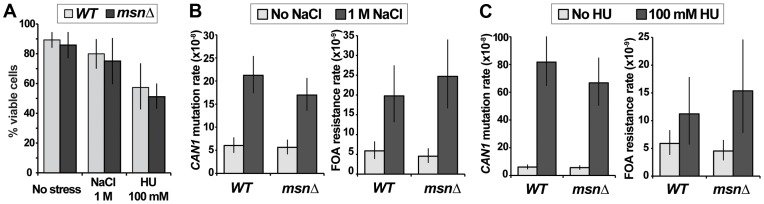
Two types of environmental stress known to cause mutagenesis do not require *MSN2* and *MSN4*. (A) Viability of the *msnΔ* strain is not significantly affected relative to that of the WT strain by growth in the presence of 1 M NaCl or 100 mM HU. Average plating efficiencies and standard deviations of at least three cultures for each strain are shown. (B) Osmotic stress in culture induces mutation or *can1* and generation of FOA^R^ mutants both in WT and *msnΔ* strains. (C) DNA replication stress induces mutation of *can1* more strongly than FOA^R^ mutagenesis, and neither process depends on *MSN2* and *MSN4*. Plotted are mutation rates and 95% confidence intervals calculated from fluctuation experiments using at least 40 cultures for canavanine resistance and at least 80 cultures for FOA resistance.

Our results indicated that under conditions of canavanine stress *MSN2* and *MSN4* could either promote or suppress mutagenesis, depending on the genetic context (e.g. presence or absence of Polζ or Ku). Several other types of environmental stress are mutagenic in yeast, in particular osmotic and DNA replication stresses [20], [21]. Both types of stress also activate the ESR, as evidenced by characteristic gene expression signatures and/or localization of Msn2 to the nucleus [1], [2], [31], [35]. To investigate whether these types of stress-induced mutagenesis also required the function of Msn2 and Msn4, we measured the rates of emergence of resistance to canavanine or 5-FOA in cells growing in the presence of osmotic stress (1M NaCl) or replication stress (100 mM hudroxyurea [HU]). These drug concentrations retarded cellular growth and reduced viability slightly (NaCl) or moderately (HU), and their effects were similar in WT and *msnΔ* cells (Figure 6A). Consistent with published reports, we observed that both types of stress were mutagenic (Figure 6B). Interestingly, while 100 mM HU induced *CAN1* mutagenesis very strongly (consistent with results in [21]), it had a much weaker effect on promoting FOA^R^ mutations (Figure 6B), suggesting that replication stress has different effects on mutagenesis at different genomic loci. Importantly, we observed that neither osmotic stress-induced mutagenesis nor replication stress-induced mutagenesis depended on *MSN2* and *MSN4* (Figure 6B). This result showed that although various stresses can activate the ESR, its role in mutagenesis is specific to certain types of stress.

### Deletion of *MSN2* and *MSN4* affects mutagenesis elicited by different types of proteotoxic stress

**Figure 7:**
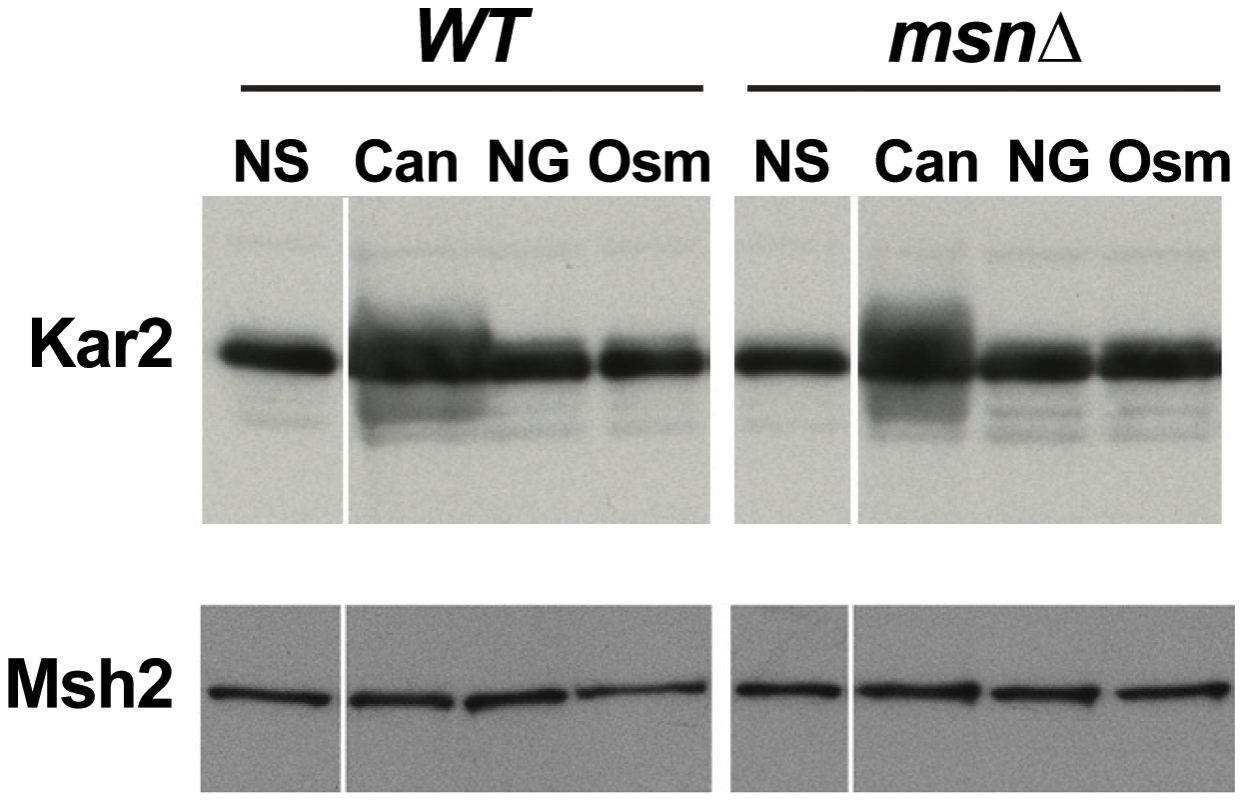
Western blot showing that the ER chaperone Kar2 is induced in response to treatment with canavanine in culture (‘Can’: cells were cultured in the presence of 100 µg/ml canavanine for 6 hours) but not to osmotic stress (‘Osm’: cells were cultured in the presence of 0.5M NaCl for 6 hours) or glucose starvation (‘NG’ = no glucose: cells were cultured in medium lacking glucose for 6 hours). Both WT and *msn2Δ msn4Δ* (*msnΔ*) strains exhibited a canavanine-dependent increase in Kar2 levels. Msh2-13xMyc served as loading control.

Although canavanine toxicity is well documented, the nature of canavanine-induced stress has not been examined. We hypothesized that canavanine induces proteotoxic stress due to accumulation of unfolded and nonfunctional proteins in which canavanines had replaced arginines. Indeed, we observed that Kar2p, an ER chaperone whose protein level is sensitive to levels of unfolded proteins [40], was increased in abundance in the presence of canavanine (Figure 7). This observation raised the possibility that Msn2-Msn4 function impinged specifically on mutagenesis caused by proteotoxic stress. There have been several other reports of proteotoxic stress promoting genome instability in yeast. For example, cells growing in the presence of another amino acid analog, *p*-Fluorophenylalanine (PFPA), showed increased rates of forward mutation at the *CAN1* locus [41]. More recently, Chen *et al.* demonstrated that various types of stress could induce aneuploidy in yeast, but that aneuploidy was induced most strongly by proteotoxic stress (specifically by an inhibitor of HSP90, radicicol) [6]. In both of these cases, the investigators hypothesized that under conditions of proteotoxic stress, increased mutagenesis and aneuploidy were due to the action of misfolded DNA repair and chromosome segregation proteins, respectively [6], [41].

**Figure 8:**
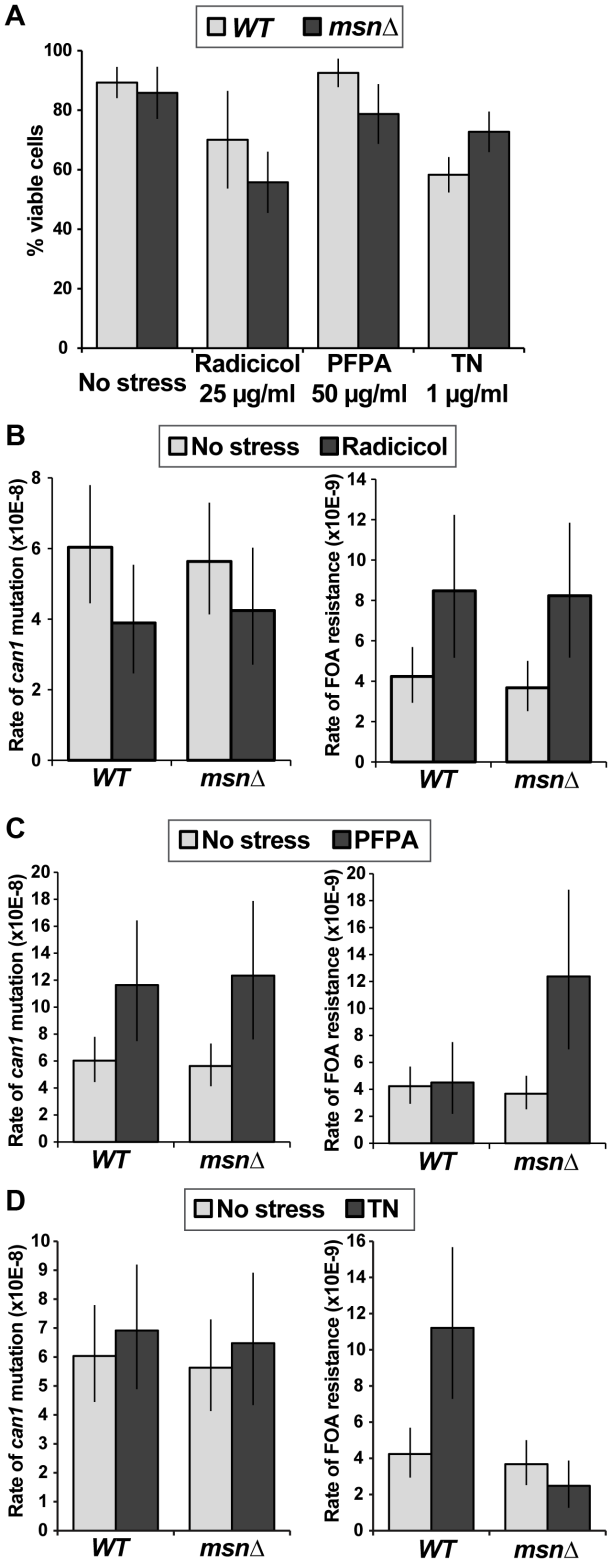
Different types of proteotoxic stress have different consequences on promoting formation canavanine-resistant versus FOA-resistant mutants and differentially involve Msn2-Msn4. (A) Graph showing cell viability of the WT and *msnΔ* strains in the presence of indicated concentrations of radicicol, PFPA, and tunicamycin (TN). (B) Mutation rates in culture to generate canavanine-resistant (left) or FOA-resistant (right) mutants in the absence and presence of 25 µg/ml radicicol. Radicicol did not induce *can1* mutagenesis but did induce FOA^R^ mutant formation in a manner that was independent of *MSN2* and *MSN4*. (C) Mutation rates in culture to generate canavanine-resistant (left) or FOA-resistant (right) mutants in the absence and presence of 50 µg/ml PFPA. PFPA induced *can1* mutagenesis by two-fold (consistent with [41]) in the WT and *msnΔ* strains. However, PFPA induced FOA^R^ mutations only in the *msnΔ* mutant, indicating that Msn2-Msn4 function to suppress PFPA-induced mutagenesis at some loci. (D) Mutation rates in culture to generate canavanine-resistant (left) or FOA-resistant (right) mutants in the absence and presence of 1 µg/ml tunicamycin (TN). Tunicamycin did not significantly affect *can1* mutagenesis, but did induce FOA^R^ mutations only in the WT strain, showing that Msn2-Msn4 can promote tunicamycin-induced mutagenesis.

To further analyze the relationship between proteotoxic stress, the ESR, and mutagenesis, we measured forward mutation rates to canavanine or FOA resistance in WT and *msnΔ* strains growing in the presence of radicicol, PFPA, or tunicamycin (a drug that inhibits protein glycosylation in the ER). We chose concentrations of the drugs that retarded cell growth and only slightly (or not at all) reduced cell viability (Figure 8A). Interestingly, we observed that each of the proteotoxic agents had a distinct effect on mutagenesis, both in terms of affected loci and in terms of Msn2-Msn4 involvement. For example, treatment with radicicol did not induce mutagenesis of *CAN1* (and may have even slightly reduced it) but did induce FOA^R^ in a manner independent of *MSN2* and *MSN4* (Figure 8B). These results showed that although radicicol is a potent inducer of aneuploidy [6], its effects on mutagenesis were mild and locus-specific. Treatment with PFPA induced mutation of *CAN1* by two-fold (consistent with data reported in [41]) in both WT and *msnΔ* strains (Figure 8C). Interestingly, PFPA induced formation of FOA^R^ mutations only in the *msnΔ* strain, showing that under these conditions, as in the *rev3Δ* and *yku80Δ* mutants, Msn2 and Msn4 must suppress a pathway that promotes mutagenesis. Finally, tunicamycin treatment had no effect on mutation of *CAN1* but induced formation of FOA^R^ mutations by over two-fold in the WT strain but not in the *msnΔ* mutant (Figure 8D). This result showed that tunicamycin-caused ER stress was mutagenic and that this mutagenesis was promoted by Msn2 and Msn4. Together, these results showed that proteotoxic stress could induce genetic instability via multiple pathways and that proteotoxic stress-induced mutagenesis was regulated by the ESR.

## Discussion

In this study, we present evidence that transcriptional activation of the ESR in the yeast *S. cerevisiae* can regulate mutagenesis elicited by several types of proteotoxic stress, including two amino acid analogs, canavanine and PFPA, and a drug that interferes with protein glycosylation, tunicamycin. The effect of the ESR was specific to proteotoxic stresses, as osmotic and DNA replication stresses elicited mutagenesis that was not affected by deletion of ESR activators, *MSN2* and *MSN4*. Moreover, Msn2 and Msn4 promoted specific types of mutation events at the *CAN1* locus, including −1 deletions in simple repeats and altered types of base pair substitutions. Two TLS polymerases, Rev1 and Rev3/Polζ, and NHEJ factor Ku promoted canavanine-induced mutagenesis in culture, and deletion of *MSN2* and *MSN4* was epistatic to *rev3Δ* and *yku80Δ* mutants. Together these results establish a previously unknown connection between a stress response pathway and specific mutagenic DNA repair and DNA damage bypass processes and provide the first example in eukaryotes of the involvement of the general stress response in mutagenesis.

Proteotoxic stress is associated with various forms of genetic instability: radicicol is a potent inducer of aneuploidy and PFPA enhances mutagenesis of *CAN1* [6], [41]. In both cases, the effect of proteotoxic stress was explained by invoking a direct role of misfolded, defective chromosome maintenance and DNA repair proteins in creating the genetic instability, without the participation of any intermediate signaling pathways [6], [41]. If this were the sole source of mutagenesis in cells experiencing proteotoxicity, then different proteotoxic agents would be expected to have similar effects. However, in this study, we observed that different types of proteotoxic stress affected different loci differently: for example, two amino acid analogs, canavanine and PFPA, have different effects on forward mutagenesis leading to FOA resistance. Furthermore, radicicol, while being a very potent inducer of aneuploidy, had relatively minor effects on mutagenesis. Even more strikingly, we observed that proteotoxic stress-induced mutagenesis was either promoted or suppressed by the ESR, arguing that proteotoxic stress induces specific signaling pathways that are regulated by Msn2-Msn4 and that culminate in the activation of mutagenic DNA repair pathways. The locus specificity may be due to the fact that mutation rates are not uniform across the genome and are influenced by local parameters such as replication timing and chromatin structure [42]–[45]; thus, proteotoxic stress and the ESR could affect at least one of these parameters. This alternative model of proteotoxic stress-induced mutagenesis is further supported by the dependency of this mutagenesis on specific DNA repair pathways, TLS and NHEJ, and by genetic interactions of the *msnΔ* mutant with DNA repair mutants, indicating that Msn2 and Msn4 functions affect DNA repair.

While our results implicate the ESR in mutagenesis, ESR-dependent mutation is not a universal consequence of every type of environmental stress. This result is consistent with recent evidence showing that both type and degree of stress affect the dynamics of Msn2 cytoplasmic-nuclear shuttling [46]. Precisely how different Msn2 nuclear dynamics correlate with downstream transcriptional changes is not yet well understood, but it is highly likely that different patterns of Msn2 nuclear entrance and exit may result in activation of different target genes. In a related fashion, different dynamics of p53 induction lead to different types of downstream responses: oscillating p53 levels activate cell cycle and DNA repair genes while constant p53 induction activates pro-apoptotic and pro-senescence genes [47]. The ESR target gene set contains several genes with known roles in chromatin structure and DNA repair that could potentially regulate mutagenesis, as well as over 100 genes with unknown functions. Undoubtedly, ongoing studies of Msn2 and Msn4 behavior in response to various stresses and of downstream effects on global transcript and protein levels will reveal the relevant targets of the ESR that regulate mutagenesis during specific types of stress.

Our results implicate two processes with known roles in mutagenesis – TLS and NHEJ. Deletion of *REV1* resulted in a partial reduction in canavanine-induced mutagenesis that was further decreased by deletion of *MSN2* and *MSN4*. In contrast, *rev3Δ* and *yku80*Δ were fully defective for canavanine-induced mutagenesis but *msnΔ*, which partially suppressed canavanine-induced mutation, was fully epistatic to these mutations. Although biochemical evidence indicates that Rev1 and Polζ can function together in TLS, with Rev1 creating a substrate for Polζ, genetic evidence has shown that their functions *in vivo* can be separable [48]. The different phenotypes of *rev1Δ* and *rev3Δ* mutants in canavanine-induced mutagenesis suggest that in this case Rev1 and Rev3 function in different branches of mutagenesis. A simple model consistent with our data is shown in Figure 5C. Canavanine induces mutagenesis through two branches, one of which is mediated by Msn2-Msn4 through Ku and Polζ while the other is inhibited by Msn and promoted by Rev1. Thus, mutation of *REV3* or *YKU80* results in inactivation of both mutagenic pathways while elimination of Msn2-Msn4 eliminates only one, even if Rev3 or Yku80 are concomitantly inactivated. No evidence currently exists for transcriptional regulation of *REV1*, *REV3*, or genes encoding the Ku complex by Msn2 and Msn4. However, our genetic data strongly suggest that the ESR regulates aspects of TLS and NHEJ. Future research will determine whether other factors in these pathways are subject to ESR regulation and/or whether this regulation may be indirect or occur at the post-translational level.

We observed that *rev3Δ* and *yku80Δ* exhibited identical phenotypes in these assays: both deletions were fully defective for canavanine-induced mutagenesis in the *MSN* strain but this defect was significantly rescued by *msnΔ*. Both Polζ and Ku have been associated with repair of DNA DSBs [30], [49], suggesting that DSBs may be an important intermediate in proteotoxic stress-induced mutagenesis. In yeast DSBs are predominantly repaired by one of two repair pathways: homologous recombination (HR) or by NHEJ [30]. HR, unlike NHEJ, uses an intact homologous sequence as template for repair and thus has been traditionally considered as the error-free DSB repair pathway. However, recent results indicate that DSB repair by HR is associated with increased mutagenesis around the DSB and that this mutagenesis is partially dependent on Polζ [50], [51]. Interestingly, DSB repair-associated mutagenesis is characterized by a distinct mutation spectrum that includes an increase in deletions in mononucleotide runs [50]. Furthermore, Lehner *et al.* recently reported that defects in NHEJ can also result in mononucleotide run instability [52]. Thus, increased deletions in mononucleotide runs observed during canavanine-induced mutagenesis of *CAN1* are consistent with DSB involvement in this mutagenic process. Interestingly, mutagenic repair of DNA DSBs also underlies stress-induced mutagenesis in *E. coli* [53], [54] and may thus represent a universal mechanism of producing genetic change during environmentally unfavorable conditions.

Our study implicates the ESR in regulating DNA repair pathways in response to proteotoxic stress in a model eukaryote and as such touches on several issues with important implications for human health. First, several lines of evidence have suggested that proteotoxic stress is an important driver of emergence of drug resistance in fungal pathogens [6], [55]. Second, tumor microenvironments are characterized by a variety of stresses, such as nutrient deprivation and hypoxia, that activate the unfolded protein response in tumor cells [56], raising the possibility that unfolded protein responses are implicated in genetic instability of cancer cells. Third, proteotoxic stresses have been recently implicated in the process of aging in worms [57], although a connection between stress and increased genetic instability of aging cells has not yet been established. To develop therapeutic approaches against stress-induced genetic instability it is essential to identify cellular pathways that promote this process. In this study we have identified several factors that promote proteotoxic stress-induced mutagenesis, including Polζ, Ku, and Msn2-Msn4. Further research into this phenomenon will reveal fundamental biological principles that underlie the roles of stress responses and DNA repair pathways in stress-induced mutagenesis, and thereby enhance the development of therapeutic approaches to combat emergence of drug resistance and genetic instability during carcinogenesis.

## Materials and Methods

### Strain construction and handling

Strains were constructed and cultured using standard yeast methods. All strains (Table S2) were derived from W303 (*leu2-3,112 trp1-1 ade2-1 his3-11,15 URA3 CAN1 RAD5*). The carrier strain for the reconstruction experiment (Figure 1C) contained two *CAN1* copies: one at the endogenous locus and one at the *rDNA* locus. The *rDNA::CAN1* gene was partially silenced but provided sufficient canavanine sensitivity to easily distinguish it from a fully canavanine-resistant strain that carried no wild type copies of *CAN1*. The strains used to analyze Msn2 loclization during canavanine stress carried endogenously expressed *MSN2-GFP*.

### The *CAN1* mutation assay

We performed the *CAN1* mutation assay almost exactly as described in [25] with some minor differences: 100 µl cultures of *CAN1* cells were grown at 23°C in synthetic complete medium containing 2% glucose and lacking arginine (SC-arg) in 96-well plates for 2 to 3 days (usually to a final concentration of approximately 10^7^ cells/culture), then spotted onto SC-arg plates containing 600 µg/mL canavanine and incubated at 23°C for 5 days. 23°C was used because originally some of the experiments included a temperature-sensitive (t.s.) mutant and to allow future comparisons to other t.s. mutants. Furthermore, incubating cells at 23°C allowed us to avoid temperature fluctuations and any potential accompanying transcriptional responses that may occur when cells are transferred from room temperature to the incubator.

For analyses of pre-plating and post-plating *can1* mutants, after 5 days of growth the plates were scanned and colony sizes were analyzed. To categorize *can1* colonies as “large” or “small”, we used Image J software (National Institutes of Health) on scanned plate images. Image J assigned a numerical value to the area of each colony and we applied a uniform threshold to categorize them as “large” or “small”. Several different threshold values were tried and the results consistently indicated that larger colonies were more likely to have arisen in culture (showed a better fit to Luria-Delbrück) while smaller colonies were more likely to have arisen after plating (showed a poorer fit to Luria-Delbrück and a better fit to Poisson). To find the best-fitting L-D distribution for a given set of data we used the MATLAB code of Lang and Murray [25] which is based on the maximum likelihood estimation method. The code was modified appropriately to find the best fitting Poisson distributions. To calculate mutation rates in culture, large colony data were analyzed using the FALCOR tool (http://www.mitochondria.org/protocols/FALCOR.html) to calculate mutation rates and 95% confidence intervals [37]. To calculate post-plating *can1* mutant frequencies, for every culture, the number of small colonies was divided by the total number of cells in the culture and these ratios were then averaged over a single experiment (72–80 cultures). For each genotype, averages and standard deviations were calculated for two to three independent experiments.

### Analyzing viability and proliferation on canavanine

To measure survival on plates containing 600 µg/ml canavanine, at different times after plating cells were micromanipulated onto canavanine-free SC plates so that viable cells could form colonies. Three to nine biological replicates were examined for each genotype at each time point. To measure the amount of post-plating proliferation, at different times after plating agar plugs containing the entire 100 µl cultures were pulled from canavanine plates, the cells were resuspended in sterile water, sonicated, and counted using a Beckman Coulter Z2 Particle Counter. Three to six biological replicates were examined for each genotype at each time point.

### Measuring FOA^R^ mutation rate in the presence or absence of stress

The FOA^R^ mutation assay was performed similarly to that for *CAN1* with a few differences. Cells were cultured in 200 µl or 250 µl of SC-arg medium +/− indicated drug concentrations at 23°C. Because the stresses retarded cell growth, the cultures were incubated for 5 to 6 days to reach 10^6^ to 10^7^ cells/well, at which point they were plated on 5-FOA plates. For canavanine-containing cultures, the wells that contained pre-existing *can1* mutations or *can1* mutations that occurred during the growth of the culture were easily identifiable because those cultures proliferated much faster and reached saturation within two or three days. Accordingly, such cultures were deemed not to be experiencing canavanine stress and were excluded from the analyses. To confirm that the cells in the slow-growing cultures had not accumulated *can1* mutations, several of these cultures were spotted onto SC-arg+600 µg/ml canavanine plates and confirmed to have only a few *can1* mutants per culture. 5-FOA-resistant colony distribution data were analyzed using the FALCOR tool (http://www.mitochondria.org/protocols/FALCOR.html) to calculate mutation rates and 95% confidence intervals [37].

### Identifying mutations at the *URA3* locus among FOA^R^ colonies

The *URA3* locus was amplified from FOA^R^ colonies using primers URA3-F3 (TGCCCAGTATTCTTAACCCAAC) and URA3-R1 (TGTTACTTGGTTCTGGCGAGG). Primer URA3-F3 was then used for sequencing by Macrogen USA. Analysis of the sequencing data revealed that in many cases the FOA^R^ colonies did not carry mutations at the *URA3* locus, suggesting that the FOA resistance was due to mutation of another gene (FigureS4). To verify that these colonies were indeed wild type for *URA3*, we performed the following phenotypic and genetic tests. We streaked 20 FOA^R^ colonies on SC-ura medium – four containing *ura3* mutations (as determined by DNA sequencing), and sixteen without detectable mutations at *URA3*. Consistent with the sequencing results, the four *ura3* mutants were unable to grow in the absence of uracil, while the sixteen *URA3* colonies were uracil prototrophs. Also, we crossed four independent *URA3* FOA^R^ colonies to a *ura3-1* strain and found each of the four FOA^R^ mutations complemented *ura3-1* for FOA resistance (the diploids were FOA^S^) and segregated independently from *ura3-1* in the cross. Thus, we concluded that in many cases FOA resistance was not due to mutation of *URA3*. We are currently investigating the identity of the non-*ura3* FOA^R^ mutations.

### Analyzing Msn2-GFP localization on canavanine plates

We examined Msn2-GFP localization using a wide-field inverted microscope (Deltavision; Applied Precision, LLC) with a charge-coupled device camera (CoolSNAP HQ; Roper Scientific), using a 100× oil-immersion objective, at 25°C and a FITC filter set to detect GFP fluorescence (Chroma, Brattleboro, VT). The transmittance was set at 10%, and the exposure time for Msn2-GFP was 200 ms, except when analyzing low fluorescence conditions (e.g. no GFP and the estradiol-inducible Msn2-GFP in the absence of estradiol) when exposure time was increased to 250 ms. To analyze whether canavanine activated the ESR, cells were taken from canavanine plates at different times after plating, resuspended in water on microscope slides, and immediately analyzed by fluorescent microscopy. To subject cells to glucose starvation, cells from the SC-arg cultures were briefly centrifuged and resuspended in SC-arg medium without glucose, incubated for one hour, then analyzed by fluorescent microscopy. Four to six z-stacks of every field were taken and projected into one image using the average pixel intensity method.

### Measuring viability of cells in culture

To measure the viability of cells in growing culture in the absence or presence of stress agents, the corresponding cultures were briefly sonicated, appropriately diluted in SC-arg medium, and plated onto YPD plates. Cell concentrations in the original cultures were obtained by using a Beckman Coulter Z2 Particle Counter.

### Analysis of Kar2 protein levels

Yeast cells were grown to mid-log phase in SC-arg medium at 23°C, then canavanine was added to a concentration of 100 µg/ml or cells were collected by filtration and transferred to SC-arg medium lacking glucose. After 6 hours, approximately 6×10^7^ cells were collected by filtration and snap-frozen at −80°C. Protein lysates were prepared form the cell pellets as described in [58]. Briefly, cells were lysed in 20% TCA using glass beads and the beads were washed twice in 5% TCA. The lysates were centrifuged, pellets resuspended in Laemmli buffer, and their pH neutralized by 2M unbuffered Tris. The resulting protein lysates were separated using 12% SDS-PAGE and probed using antibodies against Kar2 [40] and an anti-MYC antibody (Clontech) to detect Msh2-13xMyc.

### Sequencing *can1* mutations

DNA sequencing of the *CAN1* ORF was performed by Macrogen USA using primers CAN1-R1 (TGAGAATGCGAAATGGCGTG) and CAN1-R2 (TTTTGATGGCTCTTGGAACG). Statistical analyses of mutational spectra were performed and all the Fisher Exact Test (FET) p-values calculated using R open software (www.r-project.org).

## Supporting Information

Figure S1 *CAN1* and *can1-100* cells react identically to glucose withdrawal by driving Msn2-GFP into the nucleus. The images were taken 1 hour after the cells had been shifted from medium containing 2% to glucose to medium containing no glucose.
(TIF)

Figure S2 The decrease of post-plating *can1* mutation in the *msnΔ* mutant is not due a delay in emergence of post-plating mutants. The two chymographs show the timing of *can1* colonies emerging on canavanine medium. While initial (0–3 days after plating) dynamics of colony formation were identical for WT and *msnΔ* mutants, between 3 and 7 days after plating many more WT than *msnΔ* colonies appeared, reflecting an increase in post-plating mutation. Each row represents a single culture. Green bars indicate appearance of new colonies during the corresponding time period (X-axis), with variation in the shades of green reflecting the variation in number of colonies: the lightest shade of green means that one new colony arose during the corresponding time period and the darkest shade of green means that 10 or more colonies arose.
(TIF)

Figure S3 A low concentration of canavanine in culture elicits a stress response. (A) A low concentration of canavanine (2.5 µg/ml) caused increased nuclear localization of Msn2-GFP (white arrowheads). By one hour after canavanine addition, 15–20% of cells exhibited nuclear Msn2-GFP presence. (B) This low concentration of canavanine increased the doubling time of yeast cells growing in culture. The graph shows doubling times of cultures grown at 23°C in synthetic medium with and without 2.5 µg/ml canavanine. Averages and standard deviations are shown. The *msnΔ* strain grows slightly more slowly than the isogenic WT strain under both conditions.
(TIF)

Figure S4 Mutations at the *URA3* locus comprise just a fraction of spontaneous and canavanine-induced FOA^R^ mutants. This figure shows FOA^R^ rates from Figure 5B overlaid with black bars reflecting the proportion of the FOA^R^ colonies due to *ura3* mutations. Mutants were assigned to *ura3* or “other” categories by sequencing the *URA3* locus, testing uracil prototrophy, and assaying complementation/genetic linkage to *ura3-1* (for details see Materials and Methods).
(TIF)

Table S1 *can1* mutations in pre-plating and post-plating WT and *msnΔ* strains.
(PDF)

Table S2 List of strains used in this study.
(PDF)
