# Supplementary figures and images for: The Yeast Environmental Stress Response Regulates Mutagenesis Induced by Proteotoxic Stress
Source: PLoS Genet. 2013 Aug 1;9(8):e1003680. doi: 10.1371/journal.pgen.1003680 (PMC3731204; doi:10.1371/journal.pgen.1003680)

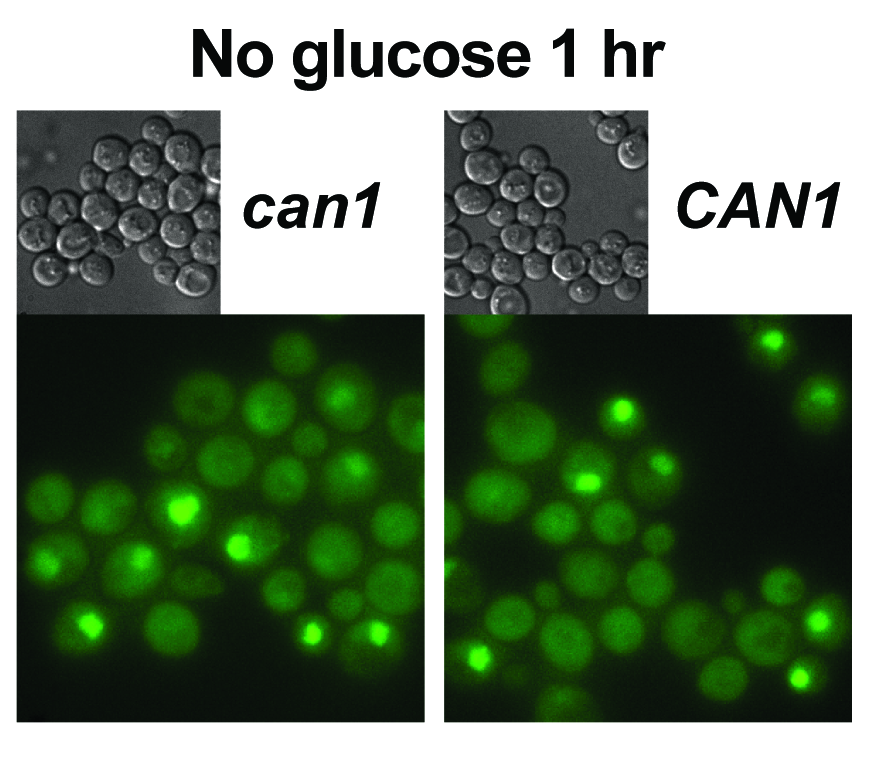

Supplement: Figure S1 — CAN1 and can1-100 cells react identically to glucose withdrawal by driving Msn2-GFP into the nucleus. The images were taken 1 hour after the cells had been shifted from medium containing 2% to glucose to medium containing no glucose. (TIF) [file pgen.1003680.s001.tif]

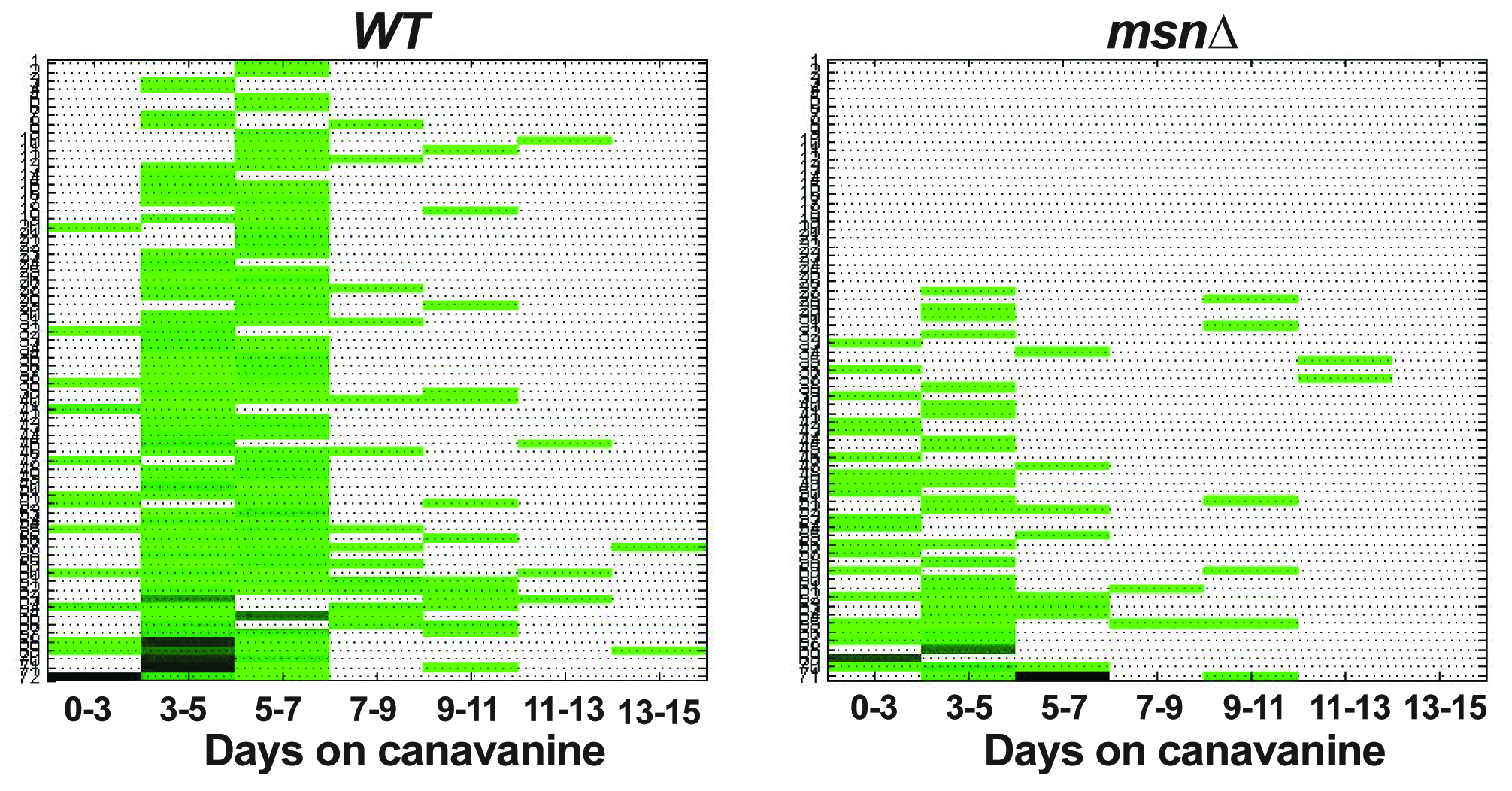

Supplement: Figure S2 — The decrease of post-plating can1 mutation in the msnΔ mutant is not due a delay in emergence of post-plating mutants. The two chymographs show the timing of can1 colonies emerging on canavanine medium. While initial (0–3 days after plating) dynamics of colony formation were identical for WT and msnΔ mutants, between 3 and 7 days after plating many more WT than msnΔ colonies appeared, reflecting an increase in post-plating mutation. Each row represents a single culture. Green bars indicate appearance of new colonies during the corresponding time period (X-axis), with variation in the shades of green reflecting the variation in number of colonies: the lightest shade of green means that one new colony arose during the corresponding time period and the darkest shade of green means that 10 or more colonies arose. (TIF) [file pgen.1003680.s002.tif]

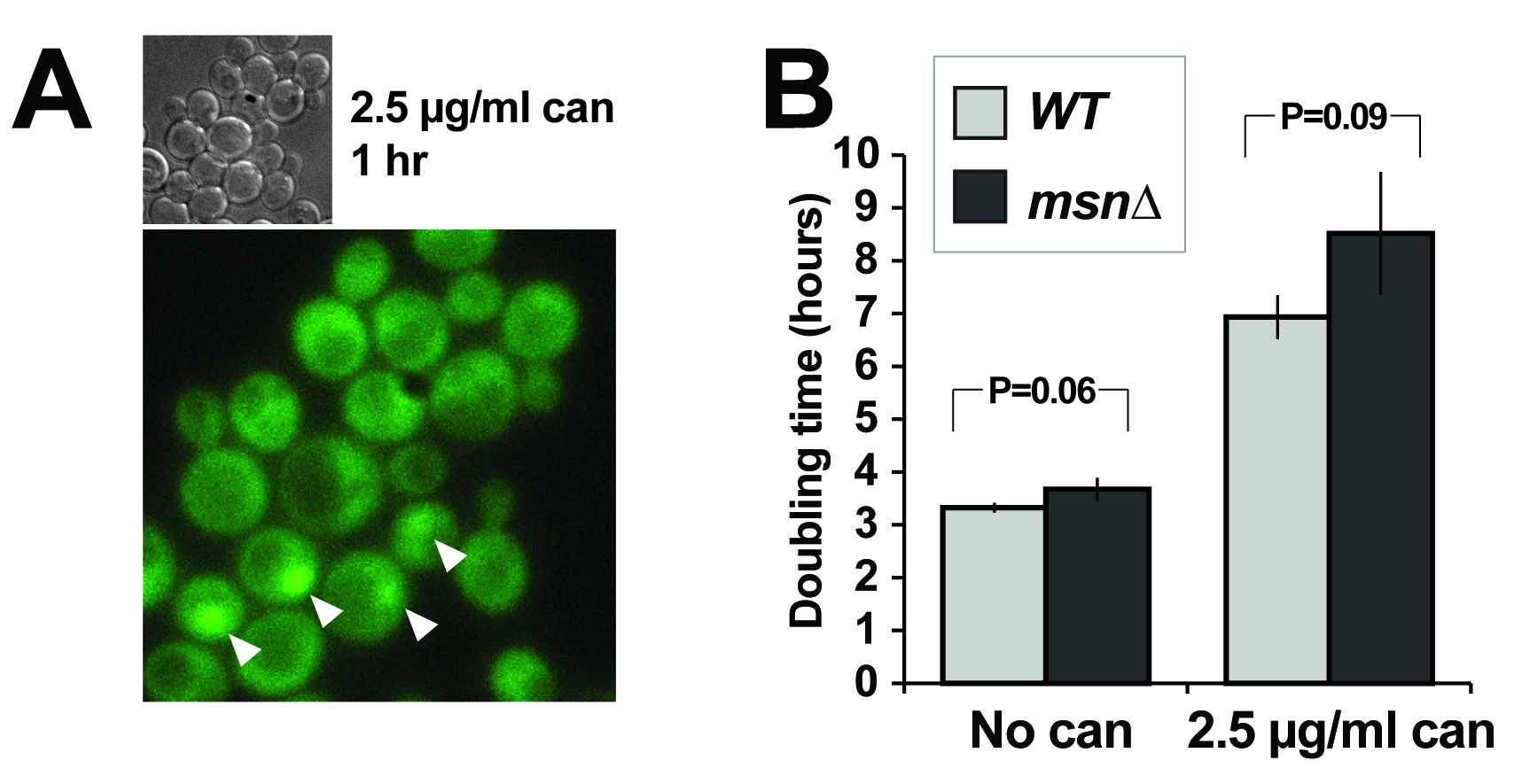

Supplement: Figure S3 — A low concentration of canavanine in culture elicits a stress response. (A) A low concentration of canavanine (2.5 µg/ml) caused increased nuclear localization of Msn2-GFP (white arrowheads). By one hour after canavanine addition, 15–20% of cells exhibited nuclear Msn2-GFP presence. (B) This low concentration of canavanine increased the doubling time of yeast cells growing in culture. The graph shows doubling times of cultures grown at 23°C in synthetic medium with and without 2.5 µg/ml canavanine. Averages and standard deviations are shown. The msnΔ strain grows slightly more slowly than the isogenic WT strain under both conditions. (TIF) [file pgen.1003680.s003.tif]

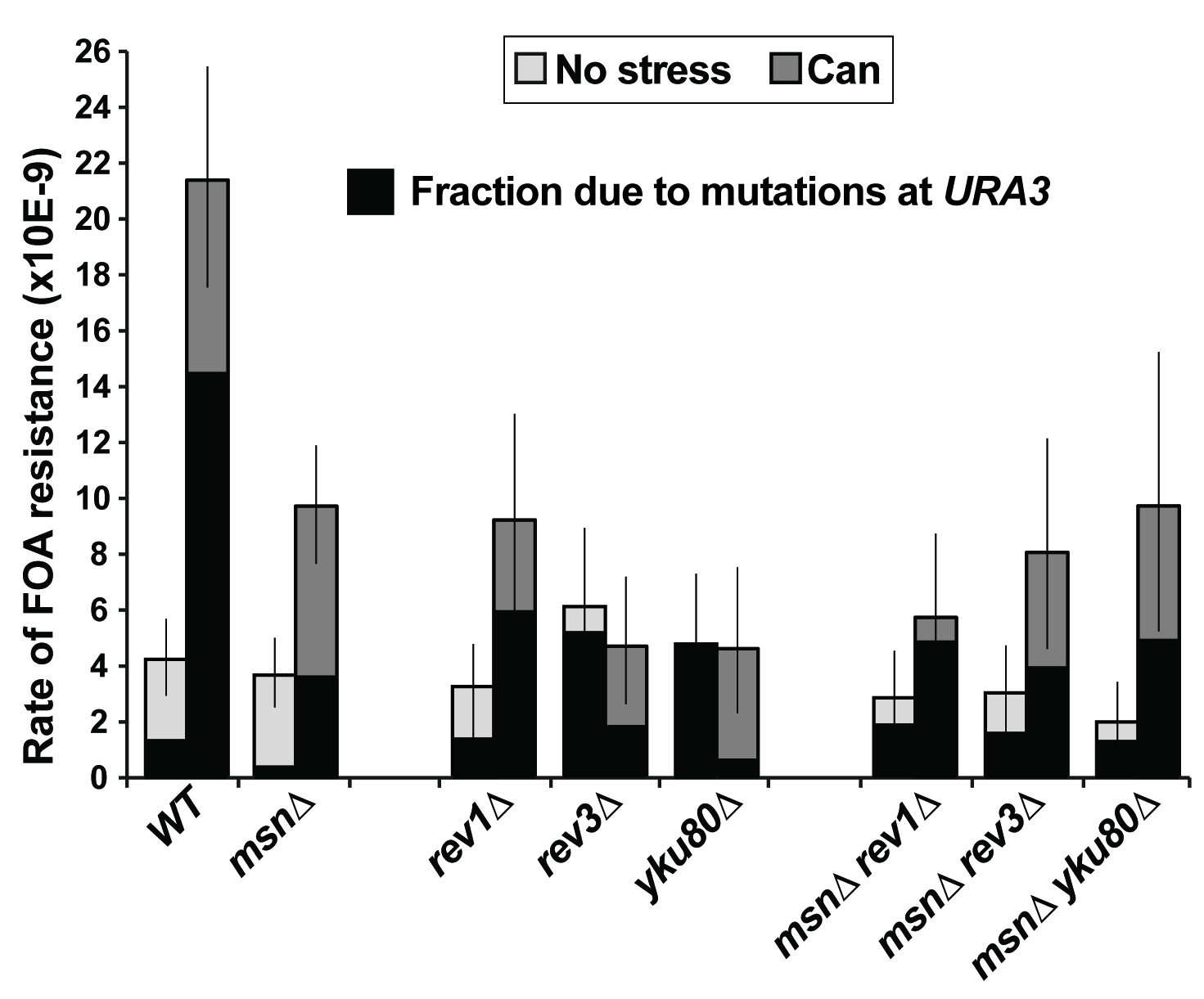

Supplement: Figure S4 — Mutations at the URA3 locus comprise just a fraction of spontaneous and canavanine-induced FOAR mutants. This figure shows FOAR rates from Figure 5B overlaid with black bars reflecting the proportion of the FOAR colonies due to ura3 mutations. Mutants were assigned to ura3 or “other” categories by sequencing the URA3 locus, testing uracil prototrophy, and assaying complementation/genetic linkage to ura3-1 (for details see Materials and Methods). (TIF) [file pgen.1003680.s004.tif]
