# Supplementary material for: The Yeast Environmental Stress Response Regulates Mutagenesis Induced by Proteotoxic Stress
Source: PLoS Genet. 2013 Aug 1;9(8):e1003680. doi: 10.1371/journal.pgen.1003680 (PMC3731204; doi:10.1371/journal.pgen.1003680)
Supplement: Table S1 — can1 mutations in pre-plating and post-plating WT and msnΔ strains. (PDF) [file pgen.1003680.s005.pdf]

## WT large

| Seq change | Location* |
|---|---|
| TCA->TGA | 113 |
| TTG-->TAG | 122 |
| AGA-->ACA | 257 |
| CTT-->CGT | 263 |
| CTT-->CCT | 263 |
| CTT->CCT | 263 |
| TAΔ (@ ATAT) | 276 |
| TGGΔ (@ TGGTGG) | 294 |
| TGGΔ (@TGGTGG) | 294 |
| GGT-->GAT | 296 |
| GTAΔ (after TGGTGG) | 299 |
| ACT->TCT | 301 |
| TTA->TAA | 329 |
| GGC-->AGC | 352 |
| GGC->GAC | 353 |
| CCA-->CTA | 356 |
| CCA-->CTA | 356 |
| T ins (@TTT) | 382 |
| ATΔ (@CATAT) | 399 |
| ATΔ (@CATAT) | 399 |
| CAG->TAG | 412 |
| TCC-->TAC | 416 |
| GAA-->AAA | 424 |
| GAA-->AAA | 424 |
| AATGGTTACATGΔ | 505 |
| GΔ (@GG) | 508 |
| GGT-->GAT | 509 |
| TAC-->TAG | 513 |
| ATG->AGG | 515 |
| AΔ (from GTATT) | 518 |
| TGG-->TAG | 521 |
| GAA-->AAA | 550 |
| GAA-->CAA | 550 |
| GAA-->GGA | 551 |
| CTT->CCT | 554 |
| TGG->TAG | 584 |
| TGG-->TGA | 585 |
| TΔ (from TTTTTT) | 620 |
| TΔ (from TTTTTT) | 620 |
| T ins (@TTTTTT) | 620 |
| ATG-->AGG | 644 |
| CCCTGT-->CTCTTT | 655 |
| GAA-->TAA | 673 |
| ATΔ (@ATAT) | 727 |
| TGT-->CGT | 733 |
| TGT-->CGT | 733 |
| TGΔ (non-repeat) | 733 |
| TAT-->TAG | 786 |
| TGG->TAG | 806 |
| TGG-->TGA | 858 |
| ACA->CCA | 886 |
| CAA->AAA | 892 |
| GGT-->GAT | 896 |
| GAA->AAA | 901 |
| GAA->AAA | 925 |
| AΔ (@AAAAA) | 964 |
| GTTΔ (from GTTGTT) | 970 |
| CGT-->TGT | 979 |
| TAC-->TCT | 995 |
| CTΔ (from CTCTCT) | 1002 |
| TCΔ (from TCTCTA) | 1003 |
| ATTΔ (from ATTATTCATT) | 1008 |
| GGA->AGA | 1018 |
| CΔ (@CC) | 1064 |
| ATΔ (@CATAT) | 1127 |
| ATΔ (@CATAT) | 1127 |
| ATΔ (@CATAT) | 1127 |
| ATΔ (@CATAT) | 1127 |
| ATΔ (@CATAT) | 1127 |
| ATΔ (@CATAT) | 1127 |
| ATΔ (@CATAT) | 1127 |
| ATΔ (@CATAT) | 1127 |
| ATΔ (@CATAT) | 1127 |
| TAC->TCC | 1184 |
| TAC-->TAG | 1185 |
| TAC->TAA | 1185 |
| TCC->TAC | 1193 |
| CGT-->GGT | 1195 |
| CGT-->CAT | 1196 |
| TAΔ (from TCTATC) | 1211 |
| AAG-->TAG | 1216 |
| AAG-->TAG | 1216 |
| GGC->GAC | 1301 |
| GGC->GAC | 1301 |
| TCTACTGGTGGTGACAΔ | 1324 |
| GGC-->GAC | 1379 |
| CΔ (from AGGCTTTTTT) | 1380 |
| AGA-->TGA | 1417 |
| CTΔ (from TCTCT) | 1449 |
| AAA->TAA | 1477 |
| 29 nt Δ | 1535 |
| CAA-->TAA | 1537 |
| CAA-->TAA | 1537 |
| CAA->TAA | 1537 |
| TΔ (from TTTT) | 1551 |

## WT small

| Seq change | Location* |
|---|---|
| AAG-->TAG | 37 |
| GAG-->TAG | 52 |
| CAA->TAA | 91 |
| GGG-->GGGG | 112 |
| TΔ (fromGGATACGT) | 191 |
| GAA->TAA | 232 |
| CAG->TAG | 238 |
| AGA-->ATA | 257 |
| AGA->AGT | 258 |
| CTT-->CGT | 263 |
| GΔ (after AAA) | 272 |
| GGT-->CGT | 295 |
| GGT->GAT | 296 |
| GGT->GAT | 299 |
| In frame Δ of 192 nt | 303 |
| GGT-->GTT | 314 |
| CTT->ATT | 316 |
| CTT-->ATT | 316 |
| GGC-->GCC | 353 |
| GGT-->GAT | 389 |
| ATΔ (from CATAT) | 400 |
| CTGTCA-->CCGGA | 405 |
| 60 nt duplication/insertion | 422 |
| ACA-->AAA | 434 |
| ACA->AAA | 434 |
| ACA->AGA | 449 |
| TΔ (@TTTT) | 465 |
| TΔ (@TTTT) | 465 |
| T ins @TTTT | 493 |
| TGG-->TAG | 521 |
| TGG->TGT | 531 |
| TGG->TGA | 531 |
| ACT-->AAT | 539 |
| GAA->CAA | 550 |
| GAA-->GTA | 551 |
| CAA->TAA | 568 |
| CAA-->TAA | 568 |
| CAA-->TAA | 577 |
| CAA->TAA | 577 |
| CAA-->TAA | 577 |
| TΔ (from TTTT) | 580 |
| TAC AAA GTT-->TAA AAA ATT T | 591 |
| TΔ (from TTTTTT) | 620 |
| TΔ (from TTTTTT) | 620 |
| TΔ (from TTTTTT) | 620 |
| TΔ (from TTTTTT) | 620 |
| AAC-->AAG | 648 |
| GGT->AGT | 670 |
| GAA->AAA | 673 |
| GAG-->GTG | 680 |
| TGG->TAG | 686 |
| GCT-->CCT | 691 |
| TGT->TGA | 735 |
| GGA-->TGA | 775 |
| TGG-->TGA | 789 |
| TGG-->TGA | 789 |
| TΔ (from GCCTGGG) | 805 |
| TGG-->TAG | 806 |
| CAA-->CGA | 892 |
| GGT-->AGT | 895 |
| GAA-->TAA | 901 |
| AΔ (from AAA) | 933 |
| CCC-->CTC | 938 |
| CAAAAAAG->AAAAAAAAA | 963 |
| GTT TTC CGT --> TTT TTC TGT | 970 |
| TΔ (from TTTT) | 974 |
| CGT->TGT | 979 |
| CGT-->TGT | 979 |
| TAC-->CAC | 994 |
| TAC->AAC | 994 |
| GGA-->AGA | 1018 |
| TΔ (@TTTT) | 1022 |
| TAC->TAA | 1035 |
| CΔ (non-repeat) | 1054 |
| TTTT-->TTTTT | 1118 |
| TΔ (@TTTT) | 1118 |
| TTG-->TAG | 1121 |
| ATΔ (from CATAT) | 1127 |
| ATΔ (from CATAT) | 1127 |
| ATΔ (from CATAT) | 1127 |
| TCT-->TTT | 1163 |
| TCA-->TAA | 1175 |
| TCC->TTC | 1193 |
| CGT--CAT | 1196 |
| AAG->TAG | 1222 |
| TCA-->TGA | 1244 |
| GGT->GAT | 1262 |
| GΔ (GG) | 1308 |
| ins: ATTAAATATCACTGGTGTTGCAGCC | 1380 |
| TGG-->TAG | 1391 |
| TGG-->TAG | 1391 |
| TCA-->TAA | 1403 |
| AGA->TGA | 1417 |
| GCT-->CCT | 1429 |
| GCT-->CCT | 1429 |
| GCT AAA TTA ATG --> GCC AAA ATA A ATC | 1476 |
| TTA-->TAA | 1481 |
| 10 nt Δ | 1510 |
| TΔ (@TT) | 1527 |
| CAA-->TAA | 1537 |
| GCTG->GGCCG | 1583 |
| 38 nt duplication/insertion | 1617 |

## msnΔ large

| Seq change | Location* |
|---|---|
| TCA-->TGA | 11 |
| TCA-->TAA | 113 |
| GAA-->TAA | 142 |
| CTT-->CCT | 263 |
| CTT-->CGT | 263 |
| AGA-->TGA | 271 |
| TAΔ (@CATAT) | 276 |
| TAΔ (@CATAT) | 276 |
| TAΔ (@CATAT) | 276 |
| TAΔ (@CATAT) | 276 |
| TAΔ (@CATAT) | 276 |
| GGT-->CGT | 298 |
| TAΔ (@TATA) | 369 |
| CTTΔ (non-repeat) | 392 |
| TΔ (non-repeat) | 428 |
| CAA-->TAA | 472 |
| TGG-->TAG | 530 |
| ACT-->GCT | 538 |
| TGG-->TGA | 585 |
| TAC-->TAG | 591 |
| TGG-->TAG | 611 |
| TΔ (@TTTTTT) | 620 |
| TΔ (@TTTTTT) | 620 |
| TGG-->TAG | 626 |
| TGG-->TAG | 626 |
| GTC-->GGC | 659 |
| GGT-->CGT | 670 |
| GGT-->GAT | 671 |
| GAA-->TAA | 673 |
| GAG-->GTG | 680 |
| GAG-->GAT | 681 |
| GGG-->CGG | 718 |
| TGT-->CGT | 733 |
| TΔ (from TTTTT) | 735 |
| TGG-->CGG | 787 |
| TGG-->CGG | 787 |
| GΔ (@GG) | 788 |
| GGT-->GAT | 809 |
| GAA-->TAA | 838 |
| CAA-->AAA | 892 |
| CAA-->TAA | 892 |
| GAA-->AAA | 901 |
| GAA-->AAA | 901 |
| GΔ (@GG) | 910 |
| GGT-->AGT | 922 |
| GGT-->AGT | 922 |
| AΔ (@AAA) | 933 |
| CCC-->ACC | 937 |
| AAΔ (@AAAAAA) | 964 |
| AAΔ @AAAAAAA | 964 |
| AΔ (@AAAAAA) | 964 |
| CGT-->CTT | 980 |
| 18 nt Δ | 1002 |
| TAΔ (from TCTCTATTA) | 1007 |
| 18 nt insertion | 1010 |
| CTT-->CGT | 1022 |
| TAC-->TAA | 1035 |
| CAA-->TAA | 1054 |
| ACΔ (@ACAC) | 1051 |
| ATΔ (@CATAT) | 1127 |
| ATΔ (@CATAT) | 1127 |
| ATΔ (@CATAT) | 1127 |
| ATΔ (@CATAT) | 1127 |
| ATΔ (@CATAT) | 1127 |
| ATΔ (@CATAT) | 1127 |
| ATΔ (@CATAT) | 1127 |
| ATΔ (@CATAT) | 1127 |
| ATΔ (@CATAT) | 1127 |
| ATΔ (@CATAT) | 1127 |
| ATΔ (@CATAT) | 1127 |
| TCT-->TAT | 1163 |
| TCA-->TAA | 1175 |
| GGT-->CGT | 1189 |
| TCC-->TTC | 1193 |
| CGT-->CTT | 1196 |
| CCA-->CGA | 1268 |
| GTT-->TTT | 1279 |
| TΔ (@TTT) | 1297 |
| GGC-->GAC | 1301 |
| 27 nt duplication | 1310 |
| GGT-->CGT | 1369 |
| AGA-->TGA | 1417 |
| 14 nt Δ | 1510 |
| ACΔ (non-repeat) | 1510 |
| 38 nt duplication | 1617 |
| GΔ (@GGGG) & TTC-->TTA | 760&684 |

## msnΔ small

| Seq change | Location* |
|---|---|
| TAC-->TAA | 48 |
| TAC-->TAA | 48 |
| TTG-->TAG | 122 |
| ATΔ (non-repeat) | 211 |
| AGA-->TGA | 256 |
| GCAAA-->TCAAT | 267 |
| AGA-->AGC | 273 |
| CAT-->TAT | 274 |
| CAT-->CGT | 275 |
| TAΔ (@CATAT) | 276 |
| TAΔ (@CATAT) | 276 |
| 27 nt deletion | 284 |
| CΔ (@CCC) | 290 |
| TCA-->TGA | 374 |
| ATΔ (@CATAT) | 375 |
| ATΔ (@CATAT) | 399 |
| ATΔ (@CATAT) | 399 |
| TCC-->TAC | 416 |
| GAA-->TAA | 424 |
| TTCAΔ | 436 |
| CCT-->CGT | 443 |
| dupl/ins: ACAT | 512 |
| GTATT-->TTAT | 516 |
| TGG-->TGC | 531 |
| TTTTG-->TTTTTT | 544 |
| CAA-->TAA | 568 |
| AΔ (@CCACT) | 600 |
| TΔ (@TTTTTT) | 620 |
| CCT-->CTT | 656 |
| GTC-->CTC | 658 |
| TAC-->TGC | 668 |
| ATA-->AGA | 728 |
| TGT-->CGT | 733 |
| TGT-->TGA | 735 |
| TGT-->TGA | 750 |
| TGG-->AGG | 787 |
| TGG-->TAG | 806 |
| TGG-->TGA | 858 |
| CAA-->TAA | 892 |
| GGT-->AGT | 922 |
| GGT-->AGT | 922 |
| CCC-->CAC | 938 |
| GTTΔ (from GTTGTT) | 970 |
| TTGΔ (@GTTGTT) | 971 |
| CGT-->CCT | 980 |
| CGT-->CTT | 980 |
| TTC-->CTC | 991 |
| TTC-->CTC | 991 |
| TAC-->TCC | 995 |
| GGC-->CGC | 1000 |
| TAC-->TAG | 1035 |
| CΔ (@CCC) | 1041 |
| TAC-->TAA | 1068 |
| TATΔ (@TATTAT) | 1086 |
| ΔAT (@CATAT) | 1127 |
| GCC-->GAC | 1166 |
| TCA-->CCA | 1174 |
| TAC-->TAA | 1185 |
| TAC-->TAA | 1185 |
| GGT-->CGT | 1189 |
| AΔ (@TTTATTT) | 1203 |
| TTG-->TAG | 1226 |
| TCA-->TGA | 1244 |
| TCA-->TGA | 1244 |
| GGT-->GAT | 1262 |
| GAA-->TAA | 1348 |
| GAA-->TAA | 1348 |
| TTA-->TGA | 1358 |
| TT ins (@TTTTTT) | 1381 |
| CAC-->CTC | 1412 |
| GCT AAA-->GCC TAA | 1476 |
| TTA-->TAA | 1481 |
| ACA-->AGA | 1511 |
| ACG-->AGG | 1520 |
| CAA-->TAA | 1537 |

*** Nucleotide of CAN1 ORF where mutation occurs or starts**
