# Supplementary material for: The Yeast Environmental Stress Response Regulates Mutagenesis Induced by Proteotoxic Stress
Source: PLoS Genet. 2013 Aug 1;9(8):e1003680. doi: 10.1371/journal.pgen.1003680 (PMC3731204; doi:10.1371/journal.pgen.1003680)
Supplement: Table S2 — List of strains used in this study. (PDF) [file pgen.1003680.s006.pdf]

Table S2. Strains used in this study.

Y3980	*MAT***a** *leu2-3,112 trp1-1 ade2-1 his3-11,15 URA3 CAN1 RAD5*
Y3981	*MAT***a** *rDNA::ADE2-CAN1 leu2-3,112 trp1-1 ade2-1 his3-11,15 URA3 CAN1 RAD5*
Y3982	*MAT***a** *yku80ΔKANMX leu2-3,112 trp1-1 ade2-1 his3-11,15 URA3 CAN1 RAD5*
Y3983	*MAT***a** *rev3ΔKANMX leu2-3,112 trp1-1 ade2-1 his3-11,15 URA3 CAN1 RAD5*
Y3986	*MAT***a** *msn2ΔKANMX msn4ΔKANMX leu2-3,112 trp1-1 ade2-1 his3-11,15 URA3 CAN1 RAD5*
Y3987	*MAT***a** *msn4Δ::KANMX MSN2-GFP CAN1 RAD5 ADE2 URA3 trp1-1 leu2-3,112 his3-11,15*
Y3988	*MAT***a** *msn4Δ::KANMX MSN2-GFP can1-100 RAD5 ADE2 URA3 TRP1 leu2-3,112 his3-11,15*
Y4090	*MAT***a** *rev1Δ::KANMX leu2-3,112 trp1-1 ade2-1 his3-11,15 URA3 CAN1 RAD5*
Y4091	*MATα yku80Δ::KANMX msn2Δ::KANMX msn4Δ::KANMX leu2-3,112 trp1-1 ade2-1 his3-11,15 URA3 CAN1 RAD5*
Y4092	*MAT***a** *rev1Δ::KANMX msn2Δ::KANMX msn4Δ::KANMX leu2-3,112 trp1-1 ade2-1 his3-11,15 URA3 CAN1 RAD5*
Y4093	*MAT***a** *rev3Δ::KANMX msn2Δ::KANMX msn4Δ::KANMX leu2-3,112 trp1-1 ade2-1 his3-11,15 URA3 CAN1 RAD5*
